# Costs of inpatient care and out-of-pocket payments for COVID-19 patients: A systematic review

**DOI:** 10.1371/journal.pone.0283651

**Published:** 2023-09-20

**Authors:** Kamal Gholipour, Sama Behpaie, Shabnam Iezadi, Akbar Ghiasi, Jafar Sadegh Tabrizi

**Affiliations:** 1 Tabriz Health Services Management Research Center, School of Management and Medical Informatics, Tabriz University of Medical Sciences, Tabriz, Iran; 2 Student Research Committee, Tabriz University of Medical Sciences, Tabriz, Iran; 3 Research Center for Emergency and Disaster Resilience, Red Crescent Society of the Islamic Republic of Iran, Tehran, Iran; 4 HEB School of Business & Administration, University of the Incarnate Word, San Antonio, Texas, United States of America; Consejo Superior de Investigaciones Cientificas (CSIC), SPAIN

## Abstract

**Objective:**

To identify the costs of hospital care for patients with COVID-19 and the amount of out-of-pocket payments.

**Methods:**

We conducted a systematic review using Scopus and WEB OF SCIENCE and PubMed databases in April 5, 2022 and then updated in January 15, 2023. English articles with no publication year restrictions were included with study designs of cost-of-illness (COI) studies, cost analyses, and observational reports (cross-sectional studies and prospective and retrospective cohorts) that calculated the patient-level cost of care for COVID-19. Costs are reported in USD with purchasing power parity (PPP) conversion in 2020. The PROSPERO registration number is CRD42022334337.

**Results:**

The results showed that the highest total cost of hospitalization in intensive care per patient was 100789 USD, which was reported in Germany, and the lowest cost was 5436.77 USD, which was reported in Romania. In the present study, in the special care department, the highest percentage of total expenses is related to treatment expenses (42.23 percent), while in the inpatient department, the highest percentage of total expenses is related to the costs of hospital beds/day of routine services (39.07 percent). The highest percentage of out-of-pocket payments was 30.65 percent, reported in China, and the lowest percentage of out-of-pocket payments was 1.12 percent, reported in Iran. The highest indirect cost per hospitalization was 16049 USD, reported in USA, and the lowest was 449.07 USD, reported in India.

**Conclusion:**

The results show that the COVID-19 disease imposed a high cost of hospitalization, mainly the cost of hospital beds/day of routine services. Studies have used different methods for calculating the costs, and this has negatively impacted the comparability costs across studies. Therefore, it would be beneficial for researchers to use a similar cost calculation model to increase the compatibility of different studies.

**Systematic review registration:** PROSPERO CRD42022334337

## Introduction

Since the onset of COVID-19 in December 2019, it is globally accounting for more than 31,930 (thousands) disability-adjusted life years until April 2021, and declines in work productivity [1]. Besides devastating health and social outcomes, COVID-19 has imposed significant economic challenges on many countries. The COVID-19 pandemic has increased the probability of entering into one of the worst recessions in history. Due to the changes in people’s lifestyles as well as demand and supply shocks, the world encountered the worst economic fallout since the Great Depression [2]. Moreover, COVID-19 infection has caused higher death and morbidity among people with pre-existing health conditions, which in turn resulted in further economic scourges [3]. The unique and unpredictable pattern of the infection, lack of adequate and standard treatment or vaccine, and unexpectedly fast spread of the infection made it a major public health emergency all over the world for some time [2]. Even though countries employed various effective public health measures to curb the spread of the infection [4], the medical and psychosocial consequences of the pandemic have been too high that they have imposed severe problems on any health system [5].

Hospitals and medical centers have also been severely affected by financial strains related to the COVID-19 pandemic [6]. During the outbreak, hospitals struggled to balance their expenditures due to the increased costs of care for COVID-19 patients, the lost revenue from the cancellation of outpatient appointments and elective procedures, the creation of new facilities such as more negative pressure rooms, high-cost of excessive overtime staff, and need for hygiene and personal protective materials [6]. Moreover, due to the high prevalence and diverse health and economic consequences of COVID-19it is crucial to study the economic impact of the disease from the perspective of patients and health system [7]. According to the cost estimate made by the American Hospital Association, the impact of the COVID-19 disease on the income of American hospitals and healthcare systems has been reported to be more than 202 billion dollars [6].

Due to the different clinical severity in patients, there is a possibility of hospitalization in general wards and intensive care units (ICU), as well as invasive and non-invasive ventilation, which has been reported in different studies. Abate et al. revealed that 32 percent of patients with COVID-19 needed hospitalization in the ICU [8]. While Wang et al. reported more than 25 percent of patients in China were admitted to the ICU [9]. Given that variables such as hospitalization in the intensive care unit and the use of invasive ventilation are effective on the costs of disease treatment. Also, due to the high spread of the disease, if the percentage of patients who need hospitalization and special care measures increases, high costs will be imposed on the health system [10, 11]. This can lead to an increase in patients’ out-of-pocket payments to receive care [12–15]. Considering the imposition of high economic costs on the health and treatment system and the limitation of health system resources, if the costs are imposed directly on the patients, due to the effect of out-of-pocket payments on access to health care services, this factor can even lead to the creation of catastrophic costs or increase the death rate of patients due to lack of access to medical services [16, 17]. Therefore, it is necessary to pay attention to the costs of COVID-19 disease and out-of-pocket payments from patients. However, a comprehensive systematic review of the literature on COVID-19 costs and patient out-of-pocket payments has not been conducted. In this regard, the present study aimed to determine the cost of inpatient care and out-of-pocket payments for COVID-19 patients, which can provide valuable information to stakeholders such as the healthcare system, policymakers, and insurers.

## Methods

We conducted this systematic review following the Preferred Reporting Items for Systematic reviews and Meta-Analyses (PRISMA) guideline [18]. The PROSPERO registration number is CRD42022334337.

### Data sources

We searched major relevant electronic databases, including Scopus, Web of Science, and Medline (via PubMed), in April 2022 and then updated in early January 2023. To have a comprehensive picture of COVID-19 cost of care, we also searched refrences of selected refrences.

### Keywords and search strategies

Three experts in healthcare management and health economics with deep experience in searching scientific electronic databases identified relevant keywords by reviewing the relevant papers, discussing with professionals on relevant topics, and using medical subject headings (mesh). We outlined search strategies specifically for each database using the Boolean operator to combine the keywords. Before conducting the full search, we employed the primary search strategy in a pilot search and modified it based on the search results. A sample search strategy for PubMed is presented in Table 1, and a description of the entire search strategy is provided as a supplementary file (S1 Table).

**Table 1:** Sample search strategy for PubMed (Search date: 1-15-2023).

Raw	Query	Results
1	(((((((((((((("2019 novel coronavirus"[Title/Abstract]) OR ("COVID19"[Title/Abstract])) OR ("COVID-19"[Title/Abstract])) OR ("COVID 2019"[Title/Abstract])) OR ("2019-novel CoV"[Title/Abstract])) OR ("SARS-cov-2"[Title/Abstract])) OR ("SARS-CoV2"[Title/Abstract])) OR ("SARSCoV2"[Title/Abstract])) OR ("SARSCoV-2"[Title/Abstract])) OR ("2019-ncov"[Title/Abstract])) OR ("coronavirus disease 2019"[Title/Abstract])) OR ("coronavirus disease-19"[Title/Abstract])) OR ("2019ncov"[Title/Abstract])) OR ("SARS coronavirus 2"[Title/Abstract])) OR ("severe acute respiratory syndrome coronavirus 2"[Title/Abstract]))) AND ((((((((((((((((((((((((((((("health expenditur*"[Title/Abstract])) OR ("health expenditur*"[MeSH Terms])) OR ("health Payment"[Title/Abstract])) OR ("health cost"[Title/Abstract])) OR ("health Spending"[Title/Abstract])) OR ("Indirect expenditure"[Title/Abstract])) OR ("Indirect cost"[Title/Abstract])) OR ("Medical direct costs"[Title/Abstract])) OR ("Non-Medical Direct Costs"[Title/Abstract])) OR ("Intangible Costs"[Title/Abstract])) OR ("Therapeutics costs"[Title/Abstract])) OR ("Treatment Costs"[Title/Abstract])) OR ("Diagnostic Costs"[Title/Abstract])) OR ("lost productivity cost"[Title/Abstract])) OR ("Cost of Illness"[Title/Abstract])) OR ("Cost of Disease"[Title/Abstract])) OR ("Illness Cost"[Title/Abstract]))	471

### Eligibility criteria

Our eligibility criteria for including articles in our systematic review were Peer-reviewed, English language, original studies, observational studies, cross-sectional studies, and retrospective/prospective articles related to the cost (direct and/or indirect costs, out-of-pocket payment) of COVID-19 treatment.

We excluded modeling studies that had not reported data about the cost of illness, conference abstracts, papers that have not reported adequate information on the method of calculating costs, as well as those calculated costs based on the information extracted about cost items from other studies.

### Data management and selection

After pooling the full search results into the EndNoteX8 program, we first removed the duplicate results. Two of the authors independently reviewed the title and abstract of the studies to identify the relevant paper. Afterward, the authors meticulously reviewed the full text of the relevant studies based on the eligibility criteria and the systematic review objectives. If there was any disagreement between the reviewers, the third author’s opinions asked for including or excluding a paper.

### Data extraction and analysis

We prepared a data extraction table based on the research questions, including information on the general characteristics of the studies, cost information, out-of-pocket payments, and factors that are attributable to cost. Before using the extraction form for data extraction in the review, it was used in a pilot of three samples, and necessary changes were made.

### General characteristics of the studies

#### Direct cost information

We categorized direct costs into separate columns for inpatient services and Intensive care unit (ICU). Seeing that in some studies there was no distinction between the costs of ICU hospitalizations and general inpatient hospitalization (although patients with different severity of disease were hospitalized), we included and reported their cost information in a column named mixed costs. Cost information included length of stay (LOS), treatment cost, diagnostic tests costs, hospital bed/day or routine service costs, and other costs.

#### Indirect cost information

Indirect costs included information on the cost of lost workdays (family member), Cost of missed workdays (patient), Cost of premature death, Presenteeism, Absenteeism, Travelling Cost, Loss of Wage/ income (Patient), and Loss of Wage (Family Member).

### Data synthesis

We reported and calculated costs in USD unit based on a Purchasing Power Parity (PPP) to be able to compare the costs in different studies across different countries. Because the majority of the papers published (25 papers) were in 2020, we considered the year 2020 for calculating PPP using CCEMG–EPPI-Centre Cost Converter’ (v.1.6 last update: 29 April 2019) considering International Monetary Fund (IMF) dataset for PPP values [19].

To provide information on total cost or average cost per person or to report the cost of each of the cost components in the desired classification as well as the amount of out-of-pocket payment per person, the currencies of other countries converted to the US dollars [20, 21]. Or the conversion rate was calculated using the total cost reported in the study, which was in the standard units of the country and dollars [13, 15]. Also, in other studies, the information on the year and the end date of the study have been used to convert the cost into US dollars [20, 22–25]. In the studies that reported the average total cost and the percentage of each cost component from the total cost, the average cost per person for each cost component was calculated and reported [10, 26–28]. In studies where the total cost was reported for all patients, the cost per person was calculated using the number of people who used the service [29]. Also, the information on patient/day and the number of patients are used to calculate the cost per person. To calculate the cost per person, Patient/day information was divided by the number of patients hospitalized in a general ward or ICU, and the patient day was calculated for each patient. Then, to calculate the daily cost per person, the obtained number from the previous calculation is multiplied by each cost component as a percentage of the total cost [25].

### Quality appraisal of the studies

Two authors (SB and KG) meticulously assessed the quality of the methods of the studies using the checklist provided by Xu et al. The checklist contains 35 questions in six domains, including: “referred to its methods as micro-costing", "separate reporting of quantity and unit cost data", "classification of transparency of cost estimates", "cost components included", "Method of quantitative data collection" and "Method of unit cost data collection". According to this checklist, if the evaluation criteria were met for any study, they scored "yes", and if the evaluation criteria were not met, the score was "no" [30]. Any disagreement between the raters was discussed and resolved with a third reviewer. S2 and S3 Tables showing the results of the quality appraisal of all included studies are presented in the attached file.

## Results

**Fig 1:**
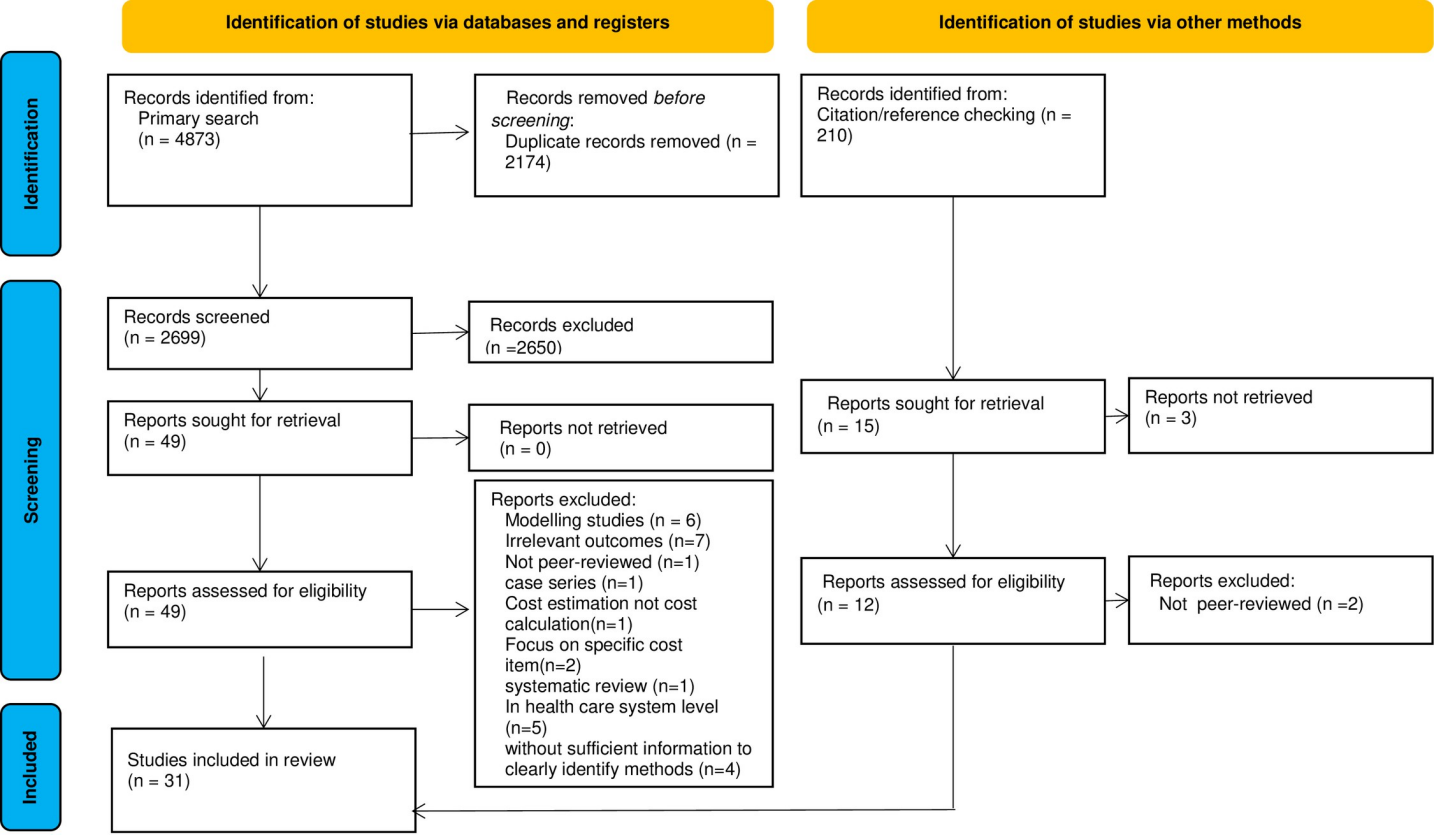
PRISMA flow diagram for systematic reviews.

Our primary search yielded 4873 results. After removing the duplicates and screening for relevant titles/abstracts, 49 papers were reviewed to select eligible studies. Finally, 31 studies met the eligibility criteria and were included in the review [10–13, 15, 16, 20–29, 31–45]. The full information on the study selection process is shown in Fig 1.

The majority of the studies (18 studies) were published in 2021.Twenty-seven studies have targeted 2020 as the baseline for calculating the costs. The studies were from low-income, average-income, and high-income countries. The included studies had conducted in Iran, China, the USA, Brazil, India, Turkey, Spain, Kenya, Romania, Ethiopia, Greece, Myanmar, Colombia, and Saudi Arabia. Most of them were from Iran (seven studies) and the US (three studies). Out of 31 studies, 11 studies have calculated costs from health system perspectives, 13 studies from health facility perspectives, two studies from patient perspectives, three studies from payer perspectives, and one from the community perspective. The costing approach in 27 studies was the bottom-up approach. Two studies used a top-down approach, and two studies were not specified. Complete information on the characteristics of the included and excluded studies can be found in the S4 and S5 Tables, respectively.

### Cost of illness for patients hospitalized in Intensive Care Units (ICU)

On average, approximately 40.1 percent of total costs were related to treatment costs, 8.7 percent to diagnostic costs, 34.2 percent to hospital bed/day or routine service costs, and 13.9 percent to other costs. The average hospitalization cost in ICU in studies that had reported cost components was 18427.68 USD. in studies that had reported only total cost per patient was 47482 USD. The highest total cost of ICU hospitalization per patient was 100,789 USD, reported in a study from the Germany, and the lowest cost of ICU hospitalization was 5436.77 USD reported in a study from Romania. Direct medical costs of inpatients with COVID-19 in the ICU are presented in Tables 2 and S6.

**Table 2:** Direct medical costs of inpatients with COVID-19 in Intensive Care Unit (ICU) (Costs were adjusted into Purchasing Power Parity (PPP) 2020).

Author (year)	Country	Treatment	Diagnostic tests	Hospital bed/day or Routine Service costs	Others	Total cost per patient	LOS
Popescu et al (2022) ***	Romania	3643.08	435.52		518.50	5436.77	14.1
**Li et al (2020)**	China	24621.14	7654.76	828.44		33137.48	16*
An et al (2022) **	China	10852.84	5830.78	3411.16	85.09	22118.61	27
Thant et al (2021) ****	Myanmar	9402.04	2858.73	313.10	6284.66	19466.57	11**
**Reddy et al. (2021)**	India	3463.12	1563.29	4860.52		12434.86	9
**Oksuz et al (2021)**	Turkey	3054.41	476.13	316.81	9870.26	13717.61	14.8
Kotwani et al (2021) ****	India	2644.89	158.50	6339.81		9143.20	10.37
**Jin et al (2019)**	China	12542.68	167.29	4716.52	7.15	17433.68	28
**Jin et al (2019)**	China	40876.73	167.29	9208.61	7.15	50223.69	42
**Ghaffari Darab et al (2021)**	Iran	3808.74	908.70	5569.20	62.40	10348.26	7*
**Miethke-Morais et al (2021)**	Brazil	2991.46	1598.14	36311.12	962.92	41864.14	
Barasa et al (2021) ****	Kenya	1838.88	486.59	6412.71	5327.09	13884.56	
**Memirie et al. (2022)**	Ethiopia	1284.14	211.29	4445.80	6030.62	11971.98	19.2
**Ebrahimipour et al (2022)**	Iran	3656.57	894.24	3986.82	143.18	8681.65	5.7
**Alvis-Zakzuk et al. (2022)**	Colombia	1366.12	700.28	4445.63	43.05	6552.21	8.1
**Khan et al. (2020)Mechanical-Ventilator Use**	Saudi Arabia					56989.97	7.93*
**Khan et al. (2020) Non-Mechanical-Ventilator Use**	Saudi Arabia					49620.56	7.93*
**Gedik (2020)**	Turkey					5646.24	14.74
**Tsai et al. (2021)**	USA					49441.00	17.1
**Ohsfeldt et al (2021)**	USA					13443.00	5
**Di Fusco et al. (2021)With ICU, but without IMV**	USA					25688.00	9.6
**Di Fusco et al. (2021) With ICU and IMV**	USA					78245.00	18.6
**Schallner et al. (2022)**	Germany					100789.00	16

Data on costs are presented as mean per patient, except for An et al. (2022), Kotwani et al. (2021), Popescu et al. (2022), and Ohsfeldt et al. (2021), which They presented as median per patient. And Barasa et al. (2020) and Jin et al. (2021) presented as unit cost.

Data on LOSare presented as mean LOS except for Li et al. (2020), Reddy et al. (2021), Khan et al. (2020), Ohsfeldt et al. (2021), which They presented as median LOS.

* LOS for each COVID-19 patient (Ward or ICU)

** LOS are the Average No for Each facility.

***In this paper, due to the small statistical population, the median costs were considered for each component, and the sum of the cost component numbers was not equal to the reported cost number.

****In this article, the total cost of components per person was not equal to the total cost per person. Reported number The total cost per person is the number reported by the article.

**Fig 2:**
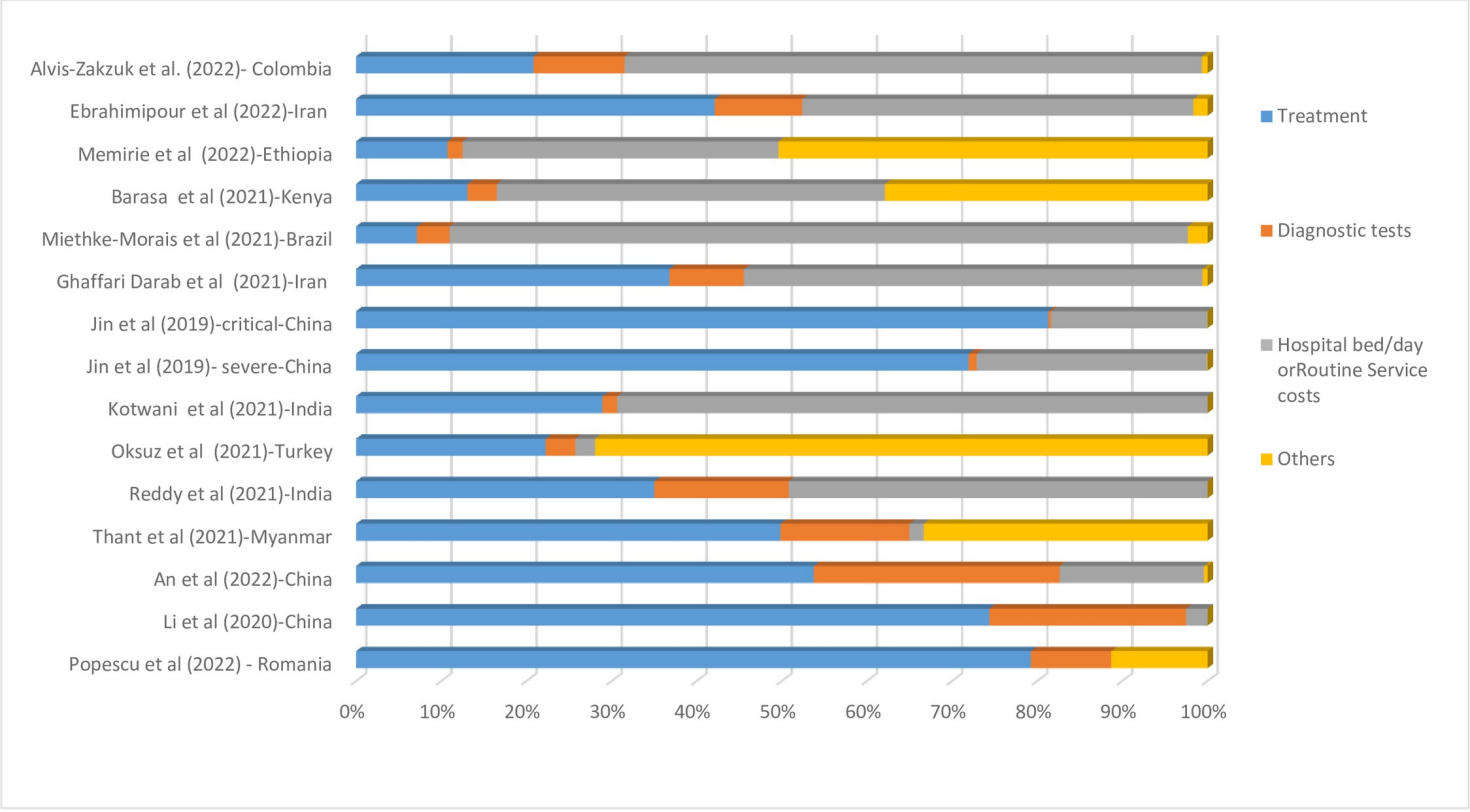
Cost drivers of hospitalization due to COVID-19 ICU per country, Data are presented as a percentage (%) of the total cost.

In a study from China, treatment costs accounted for the highest percentage of the total costs (81.38 percent), which were related to critical cases. The lowest percentage of treatment costs was in a study from Brazil, with 7.14 percent of total costs. On the other hand, a study from China reported diagnostic tests’ costs accounted for the highest percentage of total costs (26.36 percent). Another study from China reported diagnostic tests’ costs made up the lowest percentage of total costs (0.33 percent), related to critical and confirmed cases. Full information on the proportion of different elements of costs per total cost is presented in Fig 2.

### Cost of illness for patients hospitalized in general wards

Roughly 27 percent of total costs were related to treatment costs, 13 percent related to diagnostic costs, 39 percent to hospital bed/day or routine service costs, and 20 percent to other costs. The average hospitalization cost in studies reported cost components was 6299 USD. In studies that reported only the total cost per patient, it was 22880 USD, and in studies that just reported the total cost of hospitalization per patient, it was 11535 USD. The highest total cost of hospitalization in general wards per patient was 28918 USD, which was reported in a study from Saudi Arabia for cases hospitalized in general wards without Mechanical-Ventilator Use. The lowest cost of hospitalization in general wards was 1640.27 USD, which was reported in a study from Iran. Direct medical costs of inpatient care with COVID-19 in a general ward are presented in Tables 3 and S7.

**Table 3:** Direct medical costs of inpatients with COVID-19 at Ward (Costs were adjusted into Purchasing Power Parity (PPP) 2020).

Author (year)	Country	Treatment	Diagnostic tests	Hospital bed/day or Routine Service costs	Others	Total cost per patient	LOS
Thant et al (2021) ****	Myanmar	322.17	1184.33	313.09	2123.55	3937.01	11**
**Li et al (2020) Mild**	China	6730.44	2092.51	226.46		9058.48	16*
**Li et al (2020) Severe**	China	16350.02	5083.25	550.14		22005.42	16*
An et al (2022) **	China	1888.51	1004.81	1015.42	54.41	4208.15	18
**Oksuz et al (2021)**	Turkey	316.62	791.42	417.78	2236.39	3762.31	8
Kotwani et al (2021) ****	India	7.92	145.29	4226.54		4379.75	8.84
**Jin et al (2021)**	China	120.27	168.47	1549.12	7.15	1845.01	14
**Ghaffari Darab et al (2021)**	Iran	631.02	301.86	1357.98	31.20	2323.62	7*
**Miethke-Morais et al (2021)**	Brazil	685.10	467.85	11534.42	406.16	13093.43	7.71
Barasa et al (2021) ****	Kenya	1301.92	285.35	362.89	1228.01	2884.15	
**Memirie et al. (2022)-moderate**	Ethiopia	616.49	101.42	2134.37	2895.64	5747.64	9.2
**Memirie et al. (2022)-severe**	Ethiopia	752.37	123.81	2604.71	3533.28	7014.30	11.3
**Ebrahimipour et al (2022)**	Iran	690.84	168.93	753.20	27.05	1640.28	6.1
**Di Fusco et al (2021)Without ICU or IMV**	USA					14325.00	6.1
**Di Fusco et al. (2021)Without ICU, but with IMV**	USA					41769.00	12.1
**Khan et al. (2020)mechanical ventilator use GMV**	Saudi Arabia					28814.04	7.93*
**Khan et al (2020)(Non Mechanical-Ventilator Use) use GMV**	Saudi Arabia					28918.48	7.93*
**Gedik (2020)**	Turkey					1702.66	8.97
**Tsai et al. (2021)**	USA					21752.00	9.2

Data on costs are presented as mean per patient, except for An et al. (2022) and Kotwani et al. (2021), which are presented as median per patient. And in Barasa et al. (2020) and Jin et al. (2021) presented as unit cost.

Data on LOS are presented as mean LOS except for Li et al. (2020) and Khan et al. (2020), which presented as median LOS.

* LOS for each COVID-19 patient (Ward or ICU)

** LOS are the Average No for Each facility.

***In this paper, due to the small statistical population, the median costs were considered for each component, and the sum of the cost component numbers was not equal to the reported cost number.

****In this article, the total cost of components per person was not equal to the total cost per person. Reported number The total cost per person is the number reported by the article.

**Fig 3:**
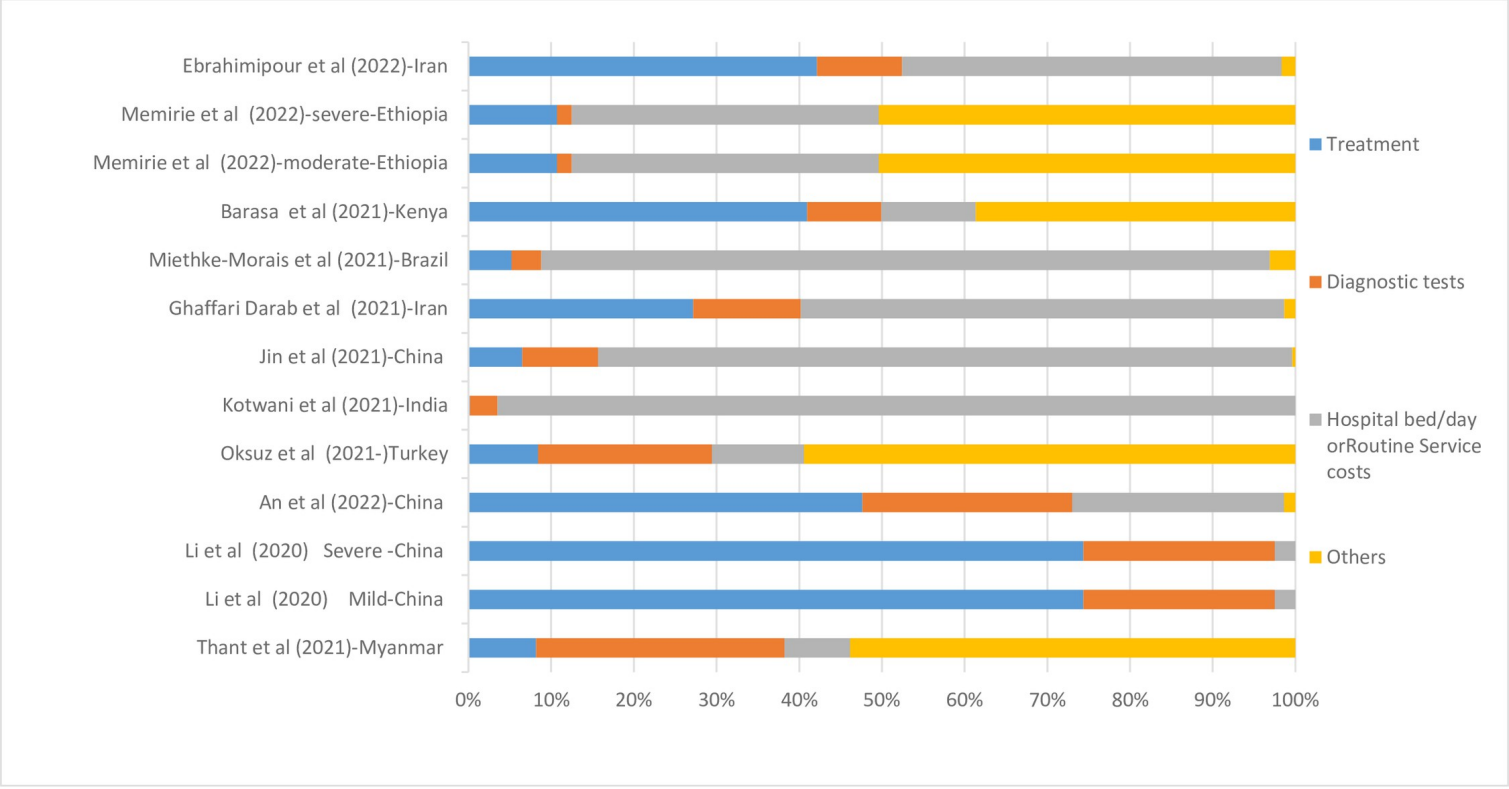
Cost drivers of hospitalization due to COVID-19 Ward per country, Data are presented as a percentage (%) of the total cost.

In a study from China, treatment costs accounted for the highest percentage of the total costs (74.29 percent). In a study from India, they reported the lowest percentage of treatment costs (0.18 percent) of total costs. On the other hand, a study from Myanmar reported that diagnostic tests’ costs accounted for the highest percentage of total costs (30.03 percent), and a study from Ethiopia reported diagnostic tests’ costs made up the lowest percentage of total costs 1.76 percent). The full information on the proportion of different elements of cists per total cost is presented in Fig 3.

### Hospitalization cost in studies in which it was not possible to separate the cost of hospitalization in the ward and the ICU (combined cost)

The average cost of hospitalization in studies in which the information on costs was categorized based on ICU hospitalization and hospitalization in wards was 18741 USD. In such studies, the highest cost was reported in a study from Spain (90685.51 USD), and the lowest cost was reported in a study from Brazil (2299.89 USD). Information on the combined direct medical costs of hospitalized patients with COVID-19 is presented in Tables 4 and S8.

**Table 4:** Combined Direct medical costs of inpatients with COVID-19 at Ward (Costs were adjusted into Purchasing Power Parity (PPP) 2020).

Author (year)	Country	Treatment	Diagnostic tests	Hospital bed/day or Routine Service costs	Others	Total cost per patient	LOS
**Santos et al (2021)**	Brazil			1948.78	351.09	2299.89	8.2
**Nakhaei et al (2021)**	Iran	373.80	277.64	940.06	109.25	1700.76	
**Haji Aghajani et al (2022)**	Iran	284.51	500.20	1256.38		2275.25	
**Haji Aghajani et al (2022)**	Iran	713.37	692.39	1522.42		3195.91	
**Hamidi Parsa et al(2022)**	Iran	53398.94		10139.04	4055.62	67593.60	
**Tabuñar et al (2021)**	Philippine	5321.78	4410.18	6020.86	2052.76	17805.57	
**Tabuñar et al (2021)**	Philippine	5890.43	5237.76	7348.72	1970.49	20447.44	
**Maltezou et al. (2021)**	Greece	70.46	697.60	5745.20	4.46	6531.83	
**Carrera-Hueso et al (2021) **	Spain	514.79	1858.65	88311.98		90685.51	8
**Damiri et al (2021)**	Iran	455.90	166.23	967.45	48.49	1636.93	1–10*
**Yusefi et al. (2022)**	Iran	4607.25	901.85	3825.40	109.90	9399.40	6.64
**Khandehroo et al (2022)**	Iran	1481.78	385.26	918.70	177.81	2963.55	
**Forrest et al (2022)**	USA					17103.00	4.8

Data on costs are presented as mean per patient, except for Haji Aghajani et al. (2022), presented as median cost per patient.

Data on LOS are presented as mean LOS in Yusefi et al. (2022), but in Carrera-Hueso et al. (2021), presented as median LOS for each COVID-19 patient (Ward or ICU) and in Damiri et al. (2021) the majority of patients (87.3 percent) had a stay length of fewer than ten days

* LOS of 87.3 percent of patients.

**Fig 4:**
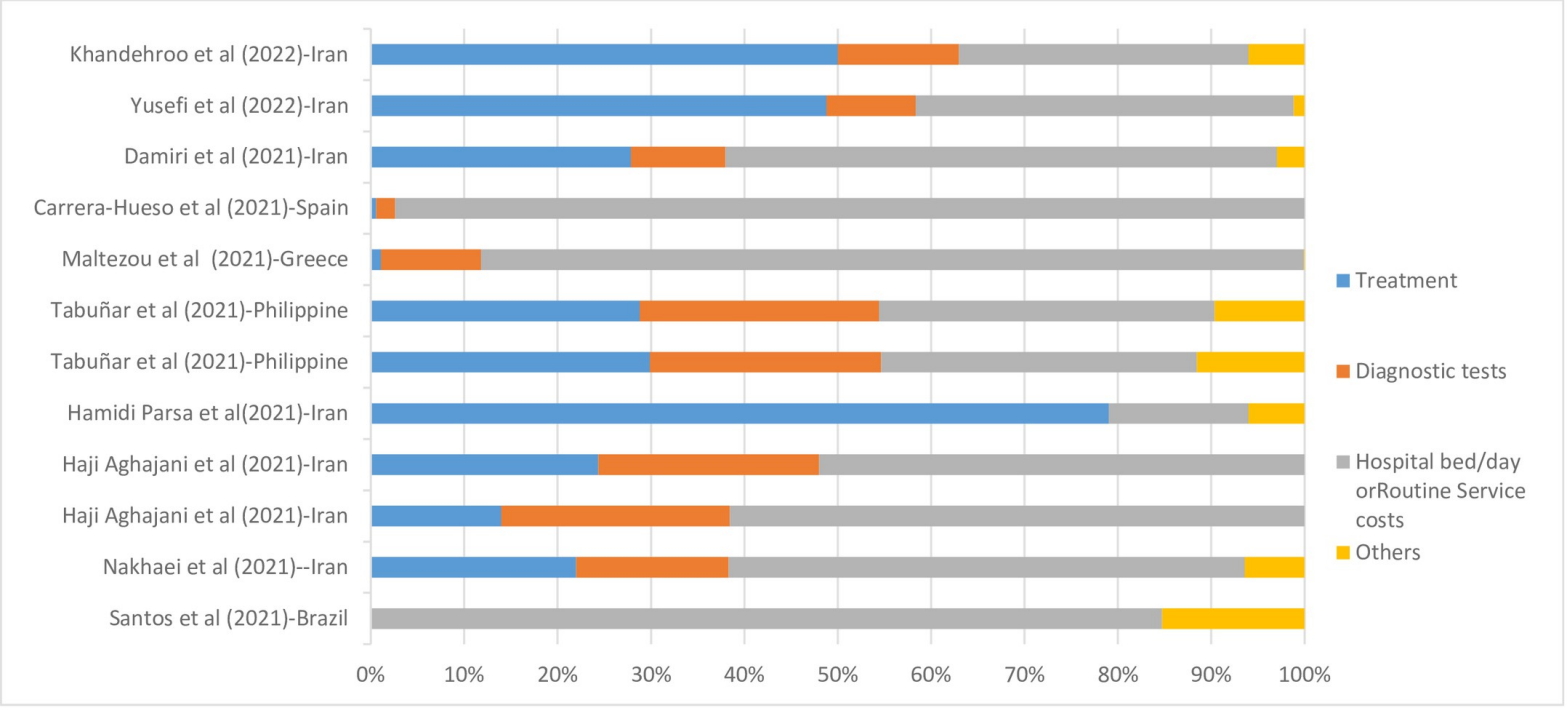
Cost drivers of hospitalization due to COVID-19 in studies in which it was not possible to separate the cost of hospitalization in the ward and the ICU, data are presented as a percentage (%) of the total cost.

In these cases, approximately 27 percent of total costs were related to treatment costs, 13 percent to diagnostic costs, 55 percent to hospital bed/day or routine service costs, and 5 percent to other costs. The full information on the proportion of different elements of costs per total cost is presented in Fig 4.

### Indirect costs

The average indirect cost of hospitalization for COVID-19 per patient was 5131.73 USD among studies reported the indirect costs. The highest indirect cost was 16049 USD per hospitalized patient in a study from USA, and the lowest indirect cost was 449.07 USD per hospitalized patient in a study from India. Table 5. Shows complete information on indirect costs of hospitalization for COVID-19.

**Table 5:** Indirect cost with COVID-19 patients (Costs were adjusted into Purchasing Power Parity (PPP) 2020).

Author (year)	Country	Cost of missed workdays (families’ patients)	Cost of missed workdays (patient)	Cost of premature death	Presenteeism	Absenteeism	Traveling Cost	Loss of Wage (Patient)	Loss of Wage (Family Member)	Total
Nakhaei et al (2021)	Iran	1089.09	1889.59	427.95						3518.40
Maltezou et al. (2021)	Greece				22.88	3719.58				3742.47
Kotwani et al (2021) *	India						50.19	211.33	187.55	449.07
Kotwani et al (2021) **	India						116.23	264.16	232.46	612.85
Ghaffari Darab et al (2021)	Iran			5293.56		830.30		294.09		6418.57
Forrest et al (2022)	USA		2076.00			13973.00				16049.00

Data on costs are presented as mean per patient, except for Kotwani et al. (2021), presented as median cost per patient.

*Hospitalization in COVID Care Centre (CCC)

** Hospitalization in ICU

**Fig 5:**
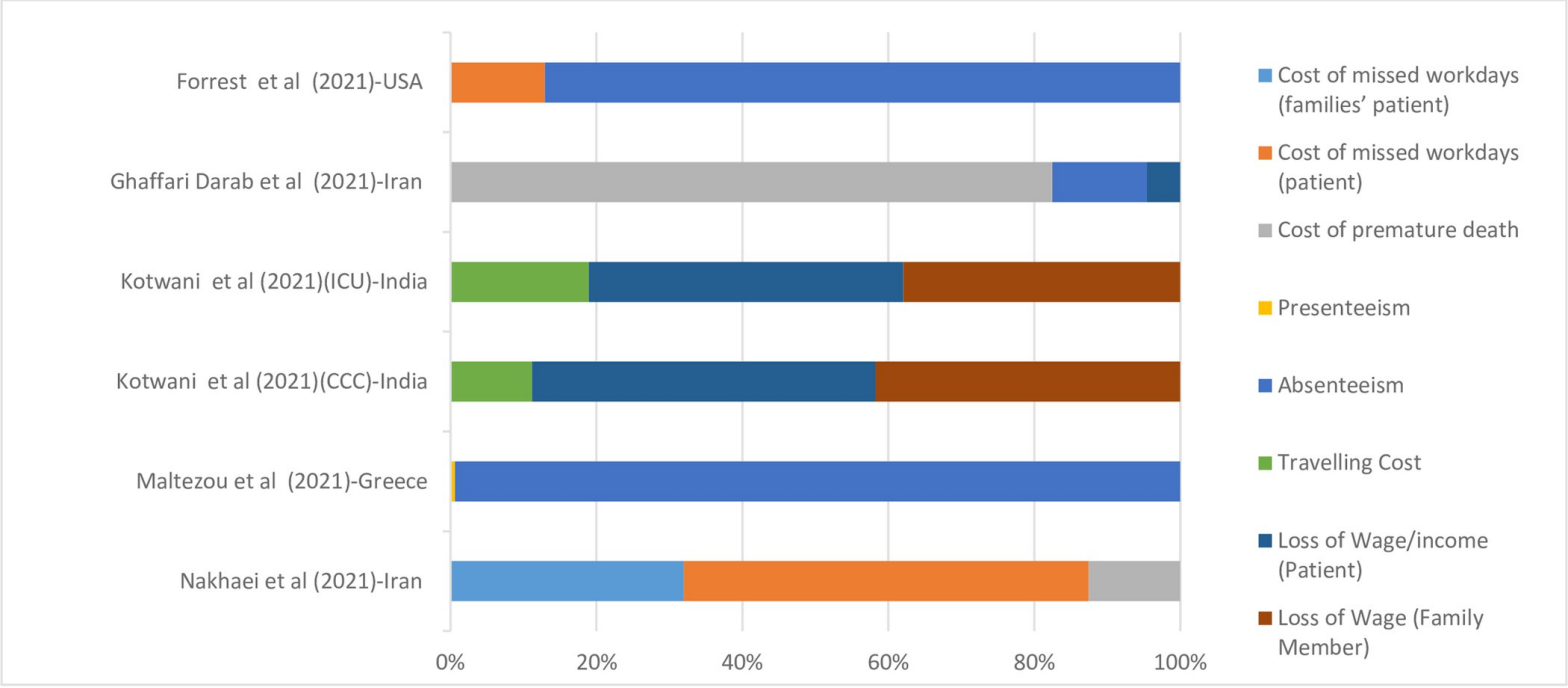
Indirect cost due to COVID-19 per country. Data are presented as a percentage (%) of the total cost.

On average, approximately 5 percent of indirect costs were related to the Cost of missed workdays (patients’ families), 11 percent Cost of missed workdays (patients), 16 percent Cost of premature death, 33 percent to Absenteeism, 5 percent to Travelling Cost, 16 percent Loss of Wage/income (Patient), and 13 percent Wage (Family Member). Fig 5 shows the indirect costs of hospitalization for COVID-19 categorized by country.

### Out of pocket

**Fig 6:**
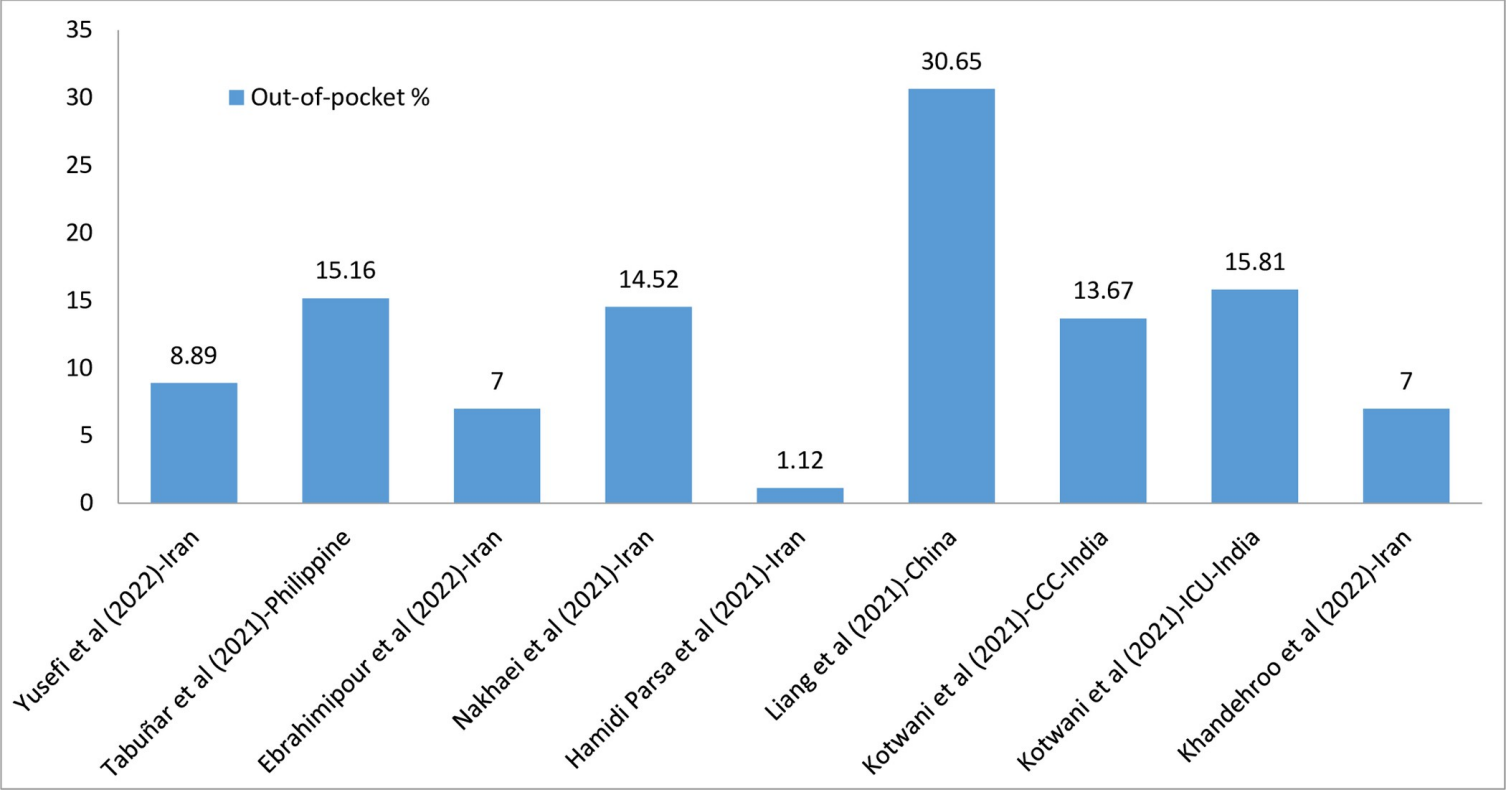
Out of pocket due to COVID-19 per country. Data are presented as a percentage (%) of the total cost.

The highest proportion of -of-pocket payment was reported in a study from China which was approximately 31 percent of the total cost. The lowest cost was reported in a study from Iran, which was about 1 percent. Fig 6 shows the percentage of the out-of-pocket payment for COVID-19 hospitalization of total cost categorized by country. Detailed information on out-of-pocket payment in each study is presented in Table 6 and Fig 6.

**Table 6:** Out of pocket due to COVID-19 per country.

Author (year)	Country	Out-of-pocket %	Total Out-of-pocket all patients	Total Out-of-pocket per patient	Total costs	Total costs per patient	Sample size
**Yusefi et al. (2022)**	Iran	8.89	92231.21	167.69	1033981.55	1879.88	550
**Tabuñar et al (2021)**	Philippine	15.16	507078.12	733.83	3345030.18	4840.85	691
**Ebrahimipour et al (2022)**	Iran	7	91575.40	30.73	1308220.00	439.00	2980
**Nakhaei et al (2021)**	Iran	14.52	285178.55	382.79	1962918.55	2634.79	745
**Hamidi Parsa (2022)**	Iran	1.12	120152.00	61.34	10717019.00	5470.65	1959
**An et al (2021)**	China	30.65	249577.92	1134.45	814325.98	3701.48	220
Kotwani et al (2021) *	India	13.67	16677.36	154.42	121997.88	1129.61	108
Kotwani et al (2021) **	India	15.81	1190.00	373.00	70746.30	2358.21	30
**Khandehroo et al (2022)**	Iran	7	592490.47	294.04	7806575.71	3874.23	2015

*Hospitalization in CCC

** Hospitalization in ICU

### COVID-19 hospitalization cost diver

Examining the cost drivers in the studies that mentioned the factors increasing the costs of hospitalization shows that underlying diseases have the most frequency in the cost drivers, and age group and severity of the disease were in the next rank (S9 Table).

## Discussion

This systematic review aimed to calculate the direct and indirect costs of hospitalization for COVID-19. Results showed significant variations in both direct and indirect COI for COVID-19 in different countries.

The results of the included studies showed that direct costs of hospitalization in the ICU and general ward were higher in the studies from the US compared to low-income and middle-income countries (LMICs). The direct costs ranged from 14325 USD to 41769 USD per patient for hospitalization at a general ward [33] and from 13443 USD to 78245 USD per patient for ICU hospitalization [33, 38]. Some studies from LMICs have reported low direct costs of hospitalization (260 USD per patient for hospitalization) for a general ward [34] and 707 USD per patient for ICU hospitalization [13]. It seems that different treatment protocols and clinical management of patients in different countries could contribute to different levels of costs. For example, in the study by Memirie et al. in Ethiopia [27], antibiotics, particularly azithromycin, were generally used to treat patients with a higher level of severity, while in the study by Li et al. in China, all patients used Chinese medicines, 69 percent of patients used Antivirus medicines and 63 percent Immunomodulators. The authors showed that 64.7 percent of the medical costs were related to these drugs, which are expensive [28].

While a few studies have provided information on components of direct costs, information on indirect costs was missed in most of the studies. For example, only one study reported the cost of missed workdays, which was 30.95 percent and 53.7 percent of the total costs for patients’ families and patients, respectively [31]. On the other hand, only one study from Greece reported presenteeism costs, which accounted for 61 percent of total costs [23]. In the studies that have reported indirect costs of COVID-19 for patients hospitalized in the ICU or a general ward, costs for Absenteeism contributed to the higher portion of total costs, which was about 994 percent of total costs in Greece [23], while in a study in Iran Absenteeism constituted the lowest proportion of total costs [12]. Although studies reveal considerable variations in the indirect cost of COVID-19, the cost of lost work hours was one of the most striking economic impacts of the pandemic. In the US, the 138 billion USD cost of lost work hours was reported during the first 12 months of the COVID-19 pandemic [46].

Out-of-pocket payments were just reported in studies from India, Iran, the Philippines, and China. In a study in China, the results showed the highest proportion of out-of-pocket expenditure (about 30.65 percent of total costs) [11]. However, the variation, even among the studies from the same country, was remarkable, and it is challenging to provide a decisive interpretation of the results. For example, two different studies from Iran have reported out-of-pocket payments made up approximately 1 percent and 14.5 percent of total costs. On the other hand, most of the studies that have reported out-of-pocket payments are from countries that do not have an integrated system to provide complete information on out-of-pocket payments. However, the detrimental economic effects of the COVID-19 pandemic on disadvantaged populations and the positive association of COVID-19 mortality with out-of-pocket expenditure highlight the importance of out-of-pocket payments and their disastrous effects [47].

The highest total cost of hospitalization at ward per patient was 41769 USD reported by Di Fusco et al. in the US [33]. In the study of Di Fusco et al., most of the participants were over 65 years old. Due to the prevalence of underlying diseases among this group of the population, the costs of this age group were higher than those under 65 years old population [33]. However, because the cost components were not specified in their study, it is not possible to compare their cost components to other studies.

The highest total cost of hospitalization in the studies without the separation of ICU hospitalization costs from other types of hospitalization was reported at 44,699.29 USD in Spain [25], in which the LOS of patients with COVID-19 was eight days. The lowest cost was 41.84 USD in Iran [25].

Different factors may contribute to variations in the level of costs in different studies. A few factors are tailored to the contexts of the study. For example, in a study in the Philippines, most of the participants were aged 61 to 70 years. Based on regulations, patients aged 60 and above receive a discount on out-of-pocket payments. As a result, the out-of-pocket expenditure was low [15]. The Source of cost data is another factor that may explain the difference in costs between studies. For instance, studies from Kenia and Myanmar have used market prices to report costs [15, 35]. Studies from Saudi Arabia and Iran, on the other hand, have used medical tariffs to report costs, and a study from Ethiopia has used medical documents in public health centers to report diagnostic costs [24, 27, 40].

Moreover, different approaches to reporting costs in different studies have resulted in variations in costs. To demonstrate, Popescu and colleagues have not included personnel reimbursement and costs of medical equipment depreciation in their calculations because, in Romania, these costs are considered healthcare costs [22]. Whereas in studies from China, personnel costs are not reported separately and are integrated with other components of healthcare costs; in studies from Myanmar, Romania, Ethiopia, India, and Brazil, personnel costs are reported separately.

### Limitations

There are some limitations in this study. First, in a few studies, only total costs were reported, and the components of costs were missing. As a result, the authors could not report the proportion of every component of costs, such as costs of treatment, diagnostic, hospital beds, regular services, and other costs, to the total costs. Second, studies have adopted different approaches to costing, which create variations in the classification of the cost components and difficulties in comparison of different studies. Third, most of the studies did not report indirect costs or just provided limited information on a few components of indirect costs. Finally, we just included English language studies in the review. Despite these limitations, we hope our study contributes to the estimation of the cost of COVID-19 hospitalization.

## Conclusion

The result of the current study shows that the COVID-19 cost of care per patient is high and these costs impose high economic burdens on the health system due to the high prevalence of the disease. In most studies, the costs of hospital beds/day and routine services have been the main drivers of costs. The difference in the calculated costs in different countries can be due to the difference in the pricing of medicines and hospital services as well as the different quality and standards of treatment in different countries. Estimating the costs of the COVID-19 disease is important from an economic point of view, and the results of this study could be used in adopting policies, planning, resource allocation, etc. There are some recommendations, such as; having uniform costing models to create uniformity and the possibility of comparison between studies. Studies should be conducted on the costs of back pain relief and its incidence in COVID-19 patients, and a review of root causes of variation in reported costs in different countries should be conducted.

## Supporting information

S1 Checklist (DOCX)

S1 Table Search strategies. (DOCX)

S2 Table Results of the risk of bias assessment for included studies. (DOCX)

S3 Table General characteristics of micro-costing studies. (DOCX)

S4 Table Characteristics of included studies (COVID-19 Inpatient care costs). (DOCX)

S5 Table Characteristics of excluded studies. (DOCX)

S6 Table Direct medical costs of inpatients with COVID-19 at Intensive Care Unit (ICU) (Costs were adjusted into Purchasing Power Parity (PPP) 2020). (DOCX)

S7 Table Direct medical costs of inpatients with COVID-19 at Ward (Costs were adjusted into Purchasing Power Parity (PPP) 2020). (DOCX)

S8 Table Combined Direct medical costs of inpatients with COVID-19 at Ward (Costs were adjusted into Purchasing Power Parity (PPP) 2020). (DOCX)

S9 Table COVID-19 hospitalization cost diver in the included study. (DOCX)
