# Supplementary material for: Costs of inpatient care and out-of-pocket payments for COVID-19 patients: A systematic review
Source: PLoS One. 2023 Sep 20;18(9):e0283651. doi: 10.1371/journal.pone.0283651 (PMC10511135; doi:10.1371/journal.pone.0283651)
Supplement: S1 Table — (DOCX) [file pone.0283651.s002.docx]

**S1 Table: Search strategies (results of the primary search)**

**Search date: 1-15-2023**

**PubMed**

| **R** | **Command** | **Strategies and keywords** | **Results** |
| --- | --- | --- | --- |
| #1 |  | **(((((((((((((("2019 novel coronavirus"[Title/Abstract]) OR ("COVID19"[Title/Abstract])) OR ("COVID-19"[Title/Abstract])) OR ("COVID 2019"[Title/Abstract])) OR ("2019-novel CoV"[Title/Abstract])) OR ("SARS-cov-2"[Title/Abstract])) OR ("SARS-CoV2"[Title/Abstract])) OR ("SARSCoV2"[Title/Abstract])) OR ("SARSCoV-2"[Title/Abstract])) OR ("2019-ncov"[Title/Abstract])) OR ("coronavirus disease 2019"[Title/Abstract])) OR ("coronavirus disease-19"[Title/Abstract])) OR ("2019ncov"[Title/Abstract])) OR ("SARS coronavirus 2"[Title/Abstract])) OR ("severe acute respiratory syndrome coronavirus 2"[Title/Abstract])** | [**314,352**](https://pubmed.ncbi.nlm.nih.gov/?term=%28%28%28%28%28%28%28%28%28%28%28%28%28%28%222019+novel+coronavirus%22%5BTitle%2FAbstract%5D%29+OR+%28%22COVID19%22%5BTitle%2FAbstract%5D%29%29+OR+%28%22COVID-19%22%5BTitle%2FAbstract%5D%29%29+OR+%28%22COVID+2019%22%5BTitle%2FAbstract%5D%29%29+OR+%28%222019-novel+CoV%22%5BTitle%2FAbstract%5D%29%29+OR+%28%22SARS-cov-2%22%5BTitle%2FAbstract%5D%29%29+OR+%28%22SARS-CoV2%22%5BTitle%2FAbstract%5D%29%29+OR+%28%22SARSCoV2%22%5BTitle%2FAbstract%5D%29%29+OR+%28%22SARSCoV-2%22%5BTitle%2FAbstract%5D%29%29+OR+%28%222019-ncov%22%5BTitle%2FAbstract%5D%29%29+OR+%28%22coronavirus+disease+2019%22%5BTitle%2FAbstract%5D%29%29+OR+%28%22coronavirus+disease-19%22%5BTitle%2FAbstract%5D%29%29+OR+%28%222019ncov%22%5BTitle%2FAbstract%5D%29%29+OR+%28%22SARS+coronavirus+2%22%5BTitle%2FAbstract%5D%29%29+OR+%28%22severe+acute+respiratory+syndrome+coronavirus+2%22%5BTitle%2FAbstract%5D%29&sort=) |
| #2 |  | **((((((((((((((((((((((((((((("health expenditur*"[Title/Abstract])) OR ("health expenditur*"[MeSH Terms])) OR ("health Payment"[Title/Abstract])) OR ("health cost"[Title/Abstract])) OR ("health Spending"[Title/Abstract])) OR ("Indirect expenditure"[Title/Abstract])) OR ("Indirect cost"[Title/Abstract])) OR ("Medical direct costs"[Title/Abstract])) OR ("Non-Medical Direct Costs"[Title/Abstract])) OR ("Intangible Costs"[Title/Abstract])) OR ("Therapeutics costs"[Title/Abstract])) OR ("Treatment Costs"[Title/Abstract])) OR ("Diagnostic Costs"[Title/Abstract])) OR ("lost productivity cost"[Title/Abstract])) OR ("Cost of Illness"[Title/Abstract])) OR ("Cost of Disease"[Title/Abstract])) OR ("Illness Cost"[Title/Abstract]))** | **45,187** |
| #3 |  | #1 AND #2 | **471** |
| Filter: English | | | |

**Search date: 1-15-2023**

**Web of Science (ISI)**

| **R** | **Command** | **Strategies and keywords** | **Results** |
| --- | --- | --- | --- |
| #1 |  | **((((((((((((((AB=("2019 novel coronavirus")) OR AB=("COVID19")) OR AB=("COVID-19")) OR AB=("COVID 2019")) OR AB=("2019-novel CoV")) OR AB=("SARS-cov-2")) OR AB=("SARS-CoV2")) OR AB=("SARSCoV2")) OR AB=("SARSCoV-2")) OR AB=("2019-ncov")) OR AB=("coronavirus disease 2019")) OR AB=("coronavirus disease-19")) OR AB=("2019ncov")) OR AB=("SARS coronavirus 2"))  OR AB=("severe acute respiratory syndrome coronavirus 2")** | 277,933 |
| #2 |  | **((((((((((((((((((((((((((((AB=("health expenditur*")) OR AB=("Direct Expenditur*")) OR AB=("Out-of-Pocket")) OR AB=("Indirect Expenditure")) OR AB=("health cost")) OR AB=("health Payment")) OR AB=("health Spending")) OR AB=("health Expens*")) OR AB=("health charge")) OR AB=("Medical Direct Costs")) OR AB=("Non-Medical Direct Costs")) OR AB=("Intangible Costs")) OR AB=(Catastrophic)) OR AB=(Catastrophe)) OR AB=(impoverish*) OR AB=("Morbidity Costs")) OR AB=("Mortality Costs")) OR AB=("inpatient care costs")) OR AB=("outpatient care costs")) OR AB=("Out of Pocket")) OR AB=("Out-of Pocket")) OR AB=("Out-of-Pocket")) OR AB=("Therapeutics costs")) OR AB=("treatment Costs")) OR AB=("Diagnostic Costs")) OR AB=("lost productivity cost")) OR AB=("Cost of Illness")) OR AB=("Cost of Disease")) OR AB=("Cost of Sickness")) OR AB=("Illness Cost")** | [92,927](https://www2.wosgs.ir/wos/woscc/summary/09bcb882-1794-4106-a880-e3fe9273d70c-6aaaabd2/relevance/1) |
| #3 |  | #1 AND #2 | [1531](https://www2.wosgs.ir/wos/woscc/summary/d1725592-fdea-4192-ae08-3824be28d313-2e836d40/relevance/1) |
| Filter: LANGUAGE: (English) | | | |

**Search date: 1-15-2023**

**SCOPUS**

| **R** | **Command** | **Strategies and keywords** | **Results** |
| --- | --- | --- | --- |
| #1 |  | TITLE-ABS-KEY ( "2019 novel coronavirus" )  OR  TITLE-ABS-KEY ( "COVID19" )  OR  TITLE-ABS-KEY ( "COVID-19" )  OR  TITLE-ABS-KEY ( "COVID 2019" )  OR  TITLE-ABS-KEY ( "2019-novel CoV" )  OR  TITLE-ABS-KEY ( "SARS-cov-2" )  OR  TITLE-ABS-KEY ( "SARS-CoV2" )  OR  TITLE-ABS-KEY ( "SARSCoV2" )  OR  TITLE-ABS-KEY ( "SARSCoV-2" )  OR  TITLE-ABS-KEY ( "2019-ncov" )  OR  TITLE-ABS-KEY ( "coronavirus disease 2019" )  OR  TITLE-ABS-KEY ( "coronavirus disease-19" )  OR  TITLE-ABS-KEY ( "2019ncov" )  OR  TITLE-ABS-KEY ( "SARS coronavirus 2" )  OR  TITLE-ABS-KEY ( "severe acute respiratory syndrome coronavirus 2" ) | 437,884 |
| #2 |  | ( TITLE-ABS-KEY ( *"catastroph*"* )  OR  TITLE-ABS-KEY ( *"health Expens*"* )  OR  TITLE-ABS-KEY ( *"Direct Expenditur*"* )  OR  TITLE-ABS-KEY ( *"health expenditur*"* )  OR  TITLE-ABS-KEY ( *"health Payment"* )  OR  TITLE-ABS-KEY ( *"health cost"* )  OR  TITLE-ABS-KEY ( *"health Spending"* )  OR  TITLE-ABS-KEY ( *"Indirect expenditure"* )  OR  TITLE-ABS-KEY ( *"Indirect cost"* )  OR  TITLE-ABS-KEY ( *"Medical direct costs"* )  OR  TITLE-ABS-KEY ( *"Non-Medical Direct Costs"* )  OR  TITLE-ABS-KEY ( *"Intangible Costs"* )  OR  TITLE-ABS-KEY ( *"catastrophic health expenditure"* )  OR  TITLE-ABS-KEY ( *"catastrophic medical expenditure"* )  OR  TITLE-ABS-KEY ( *"catastrophic health spending"* )  OR  TITLE-ABS-KEY ( *"catastrophe health expenditure"* )  OR  TITLE-ABS-KEY ( *"catastrophe medical expenditure"* )  OR  TITLE-ABS-KEY ( *"catastrophe health spending"* )  OR  TITLE-ABS-KEY ( *"impoverishment"* )  OR  TITLE-ABS-KEY ( *"Morbidity Costs"* )  OR  TITLE-ABS-KEY ( *"Mortality Costs"* )  OR  TITLE-ABS-KEY ( *"inpatient care costs"* )  OR  TITLE-ABS-KEY ( *"outpatient care costs"* )  OR  TITLE-ABS-KEY ( *"Out of Pocket"* )  OR  TITLE-ABS-KEY ( *"Out-of Pocket"* )  OR  TITLE-ABS-KEY ( *"Out-of-Pocket"* )  OR  TITLE-ABS-KEY ( *"Therapeutics costs"* )  OR  TITLE-ABS-KEY ( *"treatment Costs"* )  OR  TITLE-ABS-KEY ( *"Diagnostic Costs"* )  OR  TITLE-ABS-KEY ( *"lost productivity cost"* )  OR  TITLE-ABS-KEY ( *"Cost of Illness"* )  OR  TITLE-ABS-KEY ( *"Cost of Disease"* )  OR  TITLE-ABS-KEY ( *"Cost of Sickness"* )  OR  TITLE-ABS-KEY ( *"Illness Cost"* ) ) | 205,320 |
| #3 |  | #1 AND #2 | 2871 |
| Filter: LANGUAGE: (English) | | | |
