# Supplementary material for: Costs of inpatient care and out-of-pocket payments for COVID-19 patients: A systematic review
Source: PLoS One. 2023 Sep 20;18(9):e0283651. doi: 10.1371/journal.pone.0283651 (PMC10511135; doi:10.1371/journal.pone.0283651)
Supplement: S2 Table — (DOCX) [file pone.0283651.s003.docx]

**S2 Table: results of the risk of bias assessment for included studies**

| **Studies** | **Whether referred to its own methodology as micro-costing** | **Separate reporting of quantity and unit cost data** | **Classification of transparency of cost estimates** | **Cost components included** | **Method of quantity data collection** | **Method of unit cost data collection** | **Final Score** |
| --- | --- | --- | --- | --- | --- | --- | --- |
| (Yusefi et al., 2022) |  |  |  |  |  |  | 6 out of 6 |
| (Tabuñar et al., 2021) |  |  |  |  |  |  | 5 out of 6 |
| ([Santos](https://www.ncbi.nlm.nih.gov/pubmed/?term=dos%20Santos%20HL%5BAuthor%5D&cauthor=true&cauthor_uid=34406320) et al., 2021) |  |  |  |  |  |  | 4 out of 6 |
| (Ebrahimipour et al., 2022) |  |  |  |  |  |  | 5 out of 6 |
| (Popescu et al., 2022) |  |  |  |  |  |  | 6 out of 6 |
| (Nakhaei et al., 2021) |  |  |  |  |  |  | 5 out of 6 |
| (Hamidi Parsa et al., 2021) |  |  |  |  |  |  | 5 out of 6 |
| (Li et al., 2020) |  |  |  |  |  |  | 5 out of 6 |
| (Liang et al., 2022) |  |  |  |  |  |  | 5 out of 6 |
| (Memirie et al., 2022) |  |  |  |  |  |  | 6 out of 6 |
| ([Maltezou](https://www.ncbi.nlm.nih.gov/pubmed/?term=Maltezou%20H%5BAuthor%5D&cauthor=true&cauthor_uid=33894306) et al., 2021) |  |  |  |  |  |  | 5 out of 6 |
| (Oksuz et al., 2021) |  |  |  |  |  |  | 5 out of 6 |
| (Kotwani et al., 2021) |  |  |  |  |  |  | 5 out of 6 |
| ([Jin](https://www.ncbi.nlm.nih.gov/pubmed/?term=Jin%20H%5BAuthor%5D&cauthor=true&cauthor_uid=33551505) et al., 2020) |  |  |  |  |  |  | 5 out of 6 |
| ([Ghaffari Darab](https://www.ncbi.nlm.nih.gov/pubmed/?term=Ghaffari%20Darab%20M%5BAuthor%5D&cauthor=true&cauthor_uid=33573650) et al., 2021) |  |  |  |  |  |  | 5 out of 6 |
| ( Di Fusco et al., 2021).) |  |  |  |  |  |  | 4 out of 6 |
| ([Carrera-Hueso](https://www.ncbi.nlm.nih.gov/pubmed/?term=Carrera-Hueso%20FJ%5BAuthor%5D&cauthor=true&cauthor_uid=34734323) et al., 2021) |  |  |  |  |  |  | 5 out of 6 |
| ( Thant et al., [2021](https://bmchealthservres.biomedcentral.com/articles/10.1186/s12913-021-07394-0#article-info)) |  |  |  |  |  |  | 6 out of 6 |
| ([Reddy](https://www.ncbi.nlm.nih.gov/pubmed/?term=Reddy%20KN%5BAuthor%5D&cauthor=true&cauthor_uid=34916743) et al., 2021) |  |  |  |  |  |  | 6 out of 6 |
| ([Khan](https://www.ncbi.nlm.nih.gov/pubmed/?term=Khan%20AA%5BAuthor%5D&cauthor=true&cauthor_uid=33066327) et al., 2020) |  |  |  |  |  |  | 5 out of 6 |
| (Barasa et al., 2021) |  |  |  |  |  |  | 6 out of 6 |
| ([Miethke-Morais](https://www.ncbi.nlm.nih.gov/pubmed/?term=Miethke-Morais%20A%5BAuthor%5D&cauthor=true&cauthor_uid=34454894) et al., 2021) |  |  |  |  |  |  | 6 out of 6 |
| (Gedik., 2020) |  |  |  |  |  |  | 2 out of 6 |
| ([Tsai](https://www.ncbi.nlm.nih.gov/pubmed/?term=Tsai%20Y%5BAuthor%5D&cauthor=true&cauthor_uid=34058109) et al., 2021) |  |  |  |  |  |  | 4 out of 6 |
| ([Ohsfeldt](https://www.ncbi.nlm.nih.gov/pubmed/?term=Ohsfeldt%20RL%5BAuthor%5D&cauthor=true&cauthor_uid=34609704) et al., 2021) |  |  |  |  |  |  | 4 out of 6 |
| (Damiri et al., 2021) |  |  |  |  |  |  | 5 out of 6 |
| (Haji Aghajani et al., 2021) |  |  |  |  |  |  | 5 out of 6 |
| (Schallner et al., 2022) |  |  |  |  |  |  | 4 out of 6 |
| (Alvis-Zakzuk et al., 2021) |  |  |  |  |  |  | 5 out of 6 |
| (Khandehroo et al., 2022) |  |  |  |  |  |  | 5 out of 6 |
| (Forrest et al., 2021) |  |  |  |  |  |  | 5 out of 6 |
| Yes , No | | | | | | | |
