# Supplementary material for: Costs of inpatient care and out-of-pocket payments for COVID-19 patients: A systematic review
Source: PLoS One. 2023 Sep 20;18(9):e0283651. doi: 10.1371/journal.pone.0283651 (PMC10511135; doi:10.1371/journal.pone.0283651)
Supplement: S3 Table — (DOCX) [file pone.0283651.s004.docx]

**S*3* Table: General characteristics of micro-costing studies (1)**

|  | Characteristics | Yes | No |
| --- | --- | --- | --- |
| 1 | Whether referred to its own methodology as micro-costing |  |  |
| 2 | Separate reporting of quantity and unit cost data |  |  |
| 3 | Classification of transparency of cost estimates | | |
|  | All components of costs were described and data for both quantity and unit cost of resources were reported for each component |  |  |
|  | All components of costs were described and data for costs in each component were reported |  |  |
|  | All components of costs were described but data for costs in each component were not reported |  |  |
|  | Only scope of costing was described but components of costs were not Described |  |  |
| 4 | Cost components included | | |
|  | Personnel |  |  |
|  | Materials/supplies/consumables |  |  |
|  | Overhead |  |  |
|  | Equipment/device |  |  |
|  | Facility |  |  |
|  | Medication |  |  |
|  | Transportation |  |  |
|  | Laboratory/diagnostic/imaging test |  |  |
|  | Productivity loss |  |  |
|  | Food |  |  |
|  | Furniture |  |  |
|  | Child/elderly care |  |  |
|  | Other |  |  |
| 5 | Method of quantity data collection | | |
|  | Provider/staff interview |  |  |
|  | Hospital administrative cost/accounting database |  |  |
|  | Time-motion study |  |  |
|  | Medical chart/record review |  |  |
|  | Patient self-report |  |  |
|  | Synthesis of literature |  |  |
|  | Clinical guideline |  |  |
|  | Other |  |  |
|  | Not clear |  |  |
| 6 | Method of unit cost data collection | | |
|  | Invoice price |  |  |
|  | Hospital administrative cost/accounting database |  |  |
|  | National/regional/provincial/hospital/insurer fee schedule |  |  |
|  | Hospital/clinic/provider price catalogue |  |  |
|  | Human resources/payroll record |  |  |
|  | Other |  |  |
|  | Not clear |  |  |

1. Xu X, Lazar CM, Ruger JP. Micro-Costing In Health And Medicine: A Critical Appraisal. Health economics review. 2021;11(1):1-8.
