# Supplementary material for: Costs of inpatient care and out-of-pocket payments for COVID-19 patients: A systematic review
Source: PLoS One. 2023 Sep 20;18(9):e0283651. doi: 10.1371/journal.pone.0283651 (PMC10511135; doi:10.1371/journal.pone.0283651)
Supplement: S4 Table — (DOCX) [file pone.0283651.s005.docx]

**S4 Table. Characteristics of included studies (COVID-19 Inpatient care costs)**

| **Study ID** | **Setting, year of costing** | **Study design** | **Perspective** | **Resource Quantification** | **Data analysis** | **Data source** | **Participants/ Sample size** |
| --- | --- | --- | --- | --- | --- | --- | --- |
| Yusefi et al (2022)(17) | Iran  in a referral hospital in Shiraz, 2020 | Cross-Sectional | Health facility | Bottom-Up | Descriptive and analytical | patients’ bills were received from the hospital’s health information system (HIS) | 550 hospitalized patients  stratified sampling proportional to the sample size |
| Tabuñar et al (2021)(14) | Philippine  at the university of the Philippines-Philippine general hospital (UP-PGH) with PhilHealth coverage, 2020 | Retrospective review | Health facility | Bottom-Up | Descriptive | the Records Section, PhilHealth Unit, and the private patients' billing section  PHIC case rates that were implemented last 15 April 2020  These included medical charts admitted from the COVID charity wards and Pay floors with final ICD-10 Code Z03.8 and with additional | 691 confirmed COVID-19 adult patients were admitted at PGH |
| [Santos](https://www.ncbi.nlm.nih.gov/pubmed/?term=dos%20Santos%20HL%5BAuthor%5D&cauthor=true&cauthor_uid=34406320) et al (2021)(36) | Brazil, 2020 | observational studies | Health care system | step-down | Descriptive | the Unified Health System’s Hospital Information System (SIH-SUS) | 462,149 hospitalizations |
| Ebrahimipour et al (2022)  (11) | Iran  (including 4 coronavirus referral hospitals) in Khorasan Razavi province, 2020 | Retrospective study | Patient | Bottom-Up | Descriptive and Analytical | average cost reported by the General Office of Medical Equipment;  cost of hoteling was based on tariffs approved by the Cabinet  insurance price list portal and price coverage by the health insurance  the 2019 Book of “Relative Value of Health Services  insurance price list portal and price coverage by the health insurance  list of services and resource required by the patients, namely: (1) through examining the patients’ medical records and hospital information system; (2) evaluation of care and treatment protocols for COVID-19 issued by the Ministry of Health and Medical Education; and (3) through an interview with an expert (expert opinion). | 2980 patients  412 were admitted to ICU  2568 in a general ward |
| Popescu et al (2022)(27) | Romania  in a tertiary hospital in in the general ICU of Fundeni Clinical Institute, Bucharest, Romania, 2021 | observational studies | Health facility | Bottom-Up | Descriptive and Analytical | financial records | 36 patients diagnosed with SARS-CoV-2 infection were admitted ICU |
| Nakhaei et al (2021)(38) | Iran  The Vali-e-Asr Hospital affiliated to Birjand, 2020 | cost-of-illness study | Health facility | Bottom-Up | Descriptive and analytical | Hospital Information System(HIS) and Cost-of-illness (COI) assessment checklist. | 745 Covid-19 patients with a definite diagnosis of Covid-19 who were selected using the census method. |
| Hamidi Parsa et al (2021)(37) | Iran in Qom Province 2020 | cross-sectional | Health care system | step-down | Descriptive | Health system (IHS), health information system (HIS), and a financial software called Roozamad. | (1,959 patients) |
| Li et al ( 2020)(33) | China  In Shandong Provincial Chest Hospital, 2020 | cost analysis retrospectively | Health facility | Bottom-Up | Descriptive and analytical | electronic patient record system, , the financial management system | 70 COVID-19 patients |
| An et al (2022)(12) | China The Jiulongpo District, 2020 | cross-sectional | Health care system | Bottom-up | Descriptive and analytical | A designated hospital that received cases from 9 counties in Chongqing. | 220 inpatients with COVID-19 |
| Memirie et al (2022)(29) | Ethiopia  treatment center in Addis Ababa, the largest hospital dedicated to COVID-19 patient care in Ethiopia Ekka Kotebe center, 2020 | cost analysis retrospectively | perspective of the provider | ingredients-based approach | Descriptive | other government health facilities, finance department of the center, Ekka Kotobe finance department and World Health Organization’s (WHO) COVID-19 essential supplies forecasting tool, Ethiopian Pharmaceuticals Fund and Supply Agency | 2,543 COVID-19 cases  235 were critical  515 were severe  1,841 were moderate |
| [Maltezou](https://www.ncbi.nlm.nih.gov/pubmed/?term=Maltezou%20H%5BAuthor%5D&cauthor=true&cauthor_uid=33894306) et al (2021)(35) | Greece  (60 hospitals, 11 primary healthcare centres, four private laboratoriesand two long-term care facilities),2020 | cost-of-illness analysis | payer's perspective | bottom-up | Descriptive | Wage rates were retrieved from Law 4472/2017  Data for notified HCP with COVID-19 were retrieved from the national database, Data from HCP with COVID-19 were collected using two separate structured questionnaires. | 254 Healthcare personnel (HCP) with COVID-19 |
| Oksuz et al (2021)(21) | TurkeyA single-center, retrospective study was conducted in a tertiary hospital (Istanbul Cerrahpasa University Hospital) in İstanbul, 2020 | retrospective cohort | payer perspective | bottom-up | Descriptive and analytic statistics | Unit costs were retrieved from the Health Implementation Notification (HIN) of the SSI  the type and frequency of services used by patients as well as their utilization percentage were retrieved from the HDMS. | patients hospitalized (n = 1056)  The linear least squares regression (LSM) method was used to determine the total number of hospitalized patients during these two months.  Ward (n = 893)  ICU (n = 163) |
| Kotwani et al (2021)(15) | India  tertiary hospital in Vadodara, Gujarat, 2020 | prospective real-world study | Patient | bottom-up | Descriptive and analytic | structured questionnaire | 138 participants COVID Care Centre (CCC) incurring the cost N=108  patients admitted in COVID Intensive Care Unit (ICU) incurring the cost N=30 |
| [Jin](https://www.ncbi.nlm.nih.gov/pubmed/?term=Jin%20H%5BAuthor%5D&cauthor=true&cauthor_uid=33551505) et al (2020)(8) | China, 2019 | cost-of-illness study | Healthcare system | Bottom-up | Descriptive | resources used : information in the published literature 12,13 and clinical guidelines, 2,3  supplemented with expert opinion where necessary.  Unit cost: Shanghai: Price of healthcare services provided by health-care providers in Shanghai.  Unit cost region: multiplying the relevant cost per person data calculated for Shanghai by the healthcare industry salary index | We further divided confirmed cases into non-severe, severe and critical COVID-19, according to the disease severity  81 879 No. of confirmed cases  Survived: 28077  Died:5994 |
| [Ghaffari Darab](https://www.ncbi.nlm.nih.gov/pubmed/?term=Ghaffari%20Darab%20M%5BAuthor%5D&cauthor=true&cauthor_uid=33573650) et al (2021)(13) | Iran a referral hospital in Fars province, 2020 | cross-sectional | society perspective | bottom-up  human capital approach | Descriptive | hospital information system (HIS) | 477 COVID-19 patients who admitted to the medical centre during the 4 months  all the patients were examined through a census. |
| Di Fusco et al (2021)(26) | in the United States, 2020 | retrospective analysis | Health facility | Not mentioned | Descriptive | PHD COVID-19 Database | 173,942 hospitalized COVID-19 patients |
| [Carrera-Hueso](https://www.ncbi.nlm.nih.gov/pubmed/?term=Carrera-Hueso%20FJ%5BAuthor%5D&cauthor=true&cauthor_uid=34734323) et al (2021)(34) | SpainThe study was conducted in a 252-bed Spanish hospital that serves a catchment area of 187,258 people, 2020 | retrospective cohort study | Health facility | bottom-up | Descriptive | Unit costs for ER and ICU stays, hospitalization, and staff salaries were obtained from the official rates established for our hospital for 2020 and checked against rates for several hospitals in different regions of Spain [6]  use of individual-level data for both diagnostic tests and treatments.  Drug prices were obtained from the hospital’s pharmacy department. | 254 SARS-CoV-2-positive cohort |
| Thant et al ([2021](https://bmchealthservres.biomedcentral.com/articles/10.1186/s12913-021-07394-0#article-info))(31) | Myanmar One designated hospital and two newly established centers for COVID-19 in Yangon Region, namely Waibargi Specialist Hospital, Phaunggyi COVID-19 Treatment Center and the COVID center (Thuwana), 2020 | multicenter retrospective cost analysis | health system | the micro-costing approach  capital items | Descriptive | procurement division of MoHS, market price, input data were received from National Blood Bank, market price, unpublished survey results regarding cost analysis of selected quarantine site, swab taking and lab procedures, implemented by University of Public Health, MoHS, both medical and non-medical assets were estimated using the economic-based approach that covered both depreciation cost and opportunity cost of making the investment, administrative and financial records | Waibargi Specialist Center had treated 827 COVID-19 infected cases,  Phaunggyi Center had treated 5958 cases  Thuwanna center had treated 8162 cases  All patients: 14947 |
| [Reddy](https://www.ncbi.nlm.nih.gov/pubmed/?term=Reddy%20KN%5BAuthor%5D&cauthor=true&cauthor_uid=34916743) et al (2021)(28) | India,  in a tertiary care trust teaching hospital, 2020 | retrospective direct medical care cost analysis | Health facility | bottom- up | Descriptive and analytic | medical and billing records of patients admitted to the COVID-19 ICU | 176 patient included (122 hospital admissions, 54 direct ICU admissions) |
| [Khan](https://www.ncbi.nlm.nih.gov/pubmed/?term=Khan%20AA%5BAuthor%5D&cauthor=true&cauthor_uid=33066327) et al (2020)(22) | in the Kingdom of Saudi Arabia, 2020 | retrospective cohort | Health care system | Micro-costing | Descriptive and analytical | Health Electronic Surveillance Network (HESN)  database of the Saudi Ministry of Health (MoH) for COVID-19 patients | 1422 patients with final status (discharged alive or death) were eligible to be included in analyses |
| Barasa et al (2021)(30)  (estimate full economic costs) | Kenya, 2020 | Cost of illness | health system | bottom- up | Descriptive | input prices from a recent costing survey of 20 hospitals in Kenya and from market prices | Patients with COVID-19 with severe symptoms admitted in hospitals  Patients with COVID-19 with critical disease admitted to ICUs. 20 hospital |
| [Miethke-Morais](https://www.ncbi.nlm.nih.gov/pubmed/?term=Miethke-Morais%20A%5BAuthor%5D&cauthor=true&cauthor_uid=34454894) et al (2021)(25) | in Latin America quaternary hospital located in the city of  Sao Paulo, Brazil, 2020 | prospective, observational cohort study  a partial economic evaluation | Health facility | Micro-costing | Descriptive and analytical | Quantity based on extracted from patients’ electronic health records (EHRs)  valued according to the hospital's supply unit information | 2512 SARS-CoV-2 RT-PCR confirmed |
| Gedik (2020)(45) | Turkey  clinics or intensive care unit (ICU) of Taksim Training and Research Hospital, Istanbul, Turkey that serves with 250 beds at clinics and 38 beds at ICU,2020 | Cost analyses | Health care system | Not mentioned | Descriptive and analytical | hospital's central data processing system | 393 (76%) clinical patients (CPs) and 66 (24%) intensive care unit patients (ICUPs), |
| [Tsai](https://www.ncbi.nlm.nih.gov/pubmed/?term=Tsai%20Y%5BAuthor%5D&cauthor=true&cauthor_uid=34058109) et al (2021)(24) | United States  Medical claims for Medicare fee-for-service (FFS) beneficiaries, 2020 | Retrospective observational | Health care system | bottom- up | Descriptive and analytical statistics | Medicare fee-for-service (FFS) administrative claims data CMS FFS database | all patients: 234946  population included adults aged 65 years or older who resided in any state or the District of Columbia  hospitalization excluding death or ventilator (n= 213340)  hospitalization ventilator(n= 21606) |
| [Ohsfeldt](https://www.ncbi.nlm.nih.gov/pubmed/?term=Ohsfeldt%20RL%5BAuthor%5D&cauthor=true&cauthor_uid=34609704) et al (2021)(23) | United States, 2020 | observational | Health care system | bottom- up | Descriptive and analytical statistics | Premier Healthcare Database | 70,054 (with cost information) were included in the ICU cost analyses. |
| Damiri et al(2021)(39) | Iran, in Tehran University of Medical Sciences, 2020 | observational | Health facility | bottom- up | Descriptive and analytical statistics | patients’records | 1324 cases with a definitive diagnosis of COVID-19 |
| Haji Aghajani et al (2021)(40) | Iran, in Imam Hossein Hospital Center in Tehran, 2020 | cross-sectional | Health facility | bottom- up | Descriptive and analytical statistics | medical files of COVID-19 patients | 991 COVID-19patients |
| Schallner et al. (19) | medical centre of the University of Freiburg, Germany, between April 2020 and April 2021 | prospective single-centre observational  study | Health facility | bottom- up | Descriptive and analytical statistics | medical files of COVID-19 patients | 49 patients with COVID-19 |
| Alvis-Zakzuk et al. (20) | 23 municipalities of 12 Colombian departments (states), Colombia, 2020 | retrospective cost-of-illness study | Health facility | bottom- up | Descriptive and analytical statistics | clinical records of patients with confirmed diagnosis  of COVID-19 | 113 confirmed patients |
| Khandehroo et al (2022)-Iran (30) | Iran, Bohlool hospital, March 2020 to February 2021 | prospective single-centre observational  study | provider’s perspective | bottom- up | Descriptive and analytical statistics | medical files of COVID-19 patients | 2015 clinical confirmed COVID-19 |
| Forrest et al (2021)-USA (31) | U.S. Active Duty Army Soldiers | observational | Health care system | bottom- up | Descriptive and analytical statistics | Medical encounter data | 299 patients with COVID-19 |
