# Supplementary material for: Costs of inpatient care and out-of-pocket payments for COVID-19 patients: A systematic review
Source: PLoS One. 2023 Sep 20;18(9):e0283651. doi: 10.1371/journal.pone.0283651 (PMC10511135; doi:10.1371/journal.pone.0283651)
Supplement: S5 Table — (DOCX) [file pone.0283651.s006.docx]

# S5 Table. Characteristics of excluded studies

| **Study ID** | **Reason for exclusion** |
| --- | --- |
| Teixeira da Silva et al (2020)(1) | Modeling |
| Krylova et al (2021)(2) | Modeling |
| Jeck et al (2022)(3) | Modeling |
| Edoka et al (2021)(4) | Modeling |
| Schwendicke et al (2020)(5) | Modeling |
| Lim et al (2020)(6) | Modeling |
| Hanly et al (2022)(7) | Irrelevent outcome |
| Chua et al (2022)(8) | Irrelevent outcome |
| Chua et al (2021)(9) | Irrelevent outcome |
| Viscusi et al (2020)(10) | Irrelevent outcome |
| Garg et al (2022)(11) | Irrelevent outcome |
| Faramarzi et al (2021)(12) | Irrelevent outcome |
| Viscusi (2020)(13) | Irrelevent outcome |
| Chua et al (2021)(14) | Not Peer Reviewed |
| Banke-Thomas et al (2021)(15) | Case Seriese |
| Lopez-Valcarcel et al (2021)(16) | Cost estimation not cost calculation |
| Chua et al (2022)(17) | Focus on espesific cost item |
| Rodriguez-Gonzalez et al (2021)(18) | Focus on espesific cost item |
| Almalki et al (2021)(19) | Systematic Review |
| Nascimento et al (2022)(20) | In health care system level |
| Hartman et al (2022)(21) | In health care system level |
| Torres-Rueda et al (2021)(22) | In health care system level |
| Fatoye et al (2021)(23) | In health care system level |
| Araja et al (2021)(24) | In health care system level |
| Romero et al (2020)(25) | Wthout sufficient information to clearly identify methods |
| Thomas et al (2022) (26) | Wthout sufficient information to estimate OOP |
| Chua et al. (2021) (27) | Wthout sufficient information to estimate OOP |
| Chua et al. (2021) (28) | Wthout sufficient information to estimate OOP |

References:

1. Teixeira da Silva JA, Tsigaris P. Estimating worldwide costs of premature mortalities caused by COVID-19. Journal of Health Research. 2020;35(4):353-8.

2. Krylova O, Krasheninnikov A, Mamontova E, Tananakina G, Belyakova D. Pharmacoeconomic analysis of treatment regimens for coronavirus infection coronavirus disease-19. Open Access Macedonian Journal of Medical Sciences. 2021;9:1182-9.

3. Jeck J, Jakobs F, Kron A, Franz J, Cornely OA, Kron F. A cost of illness study of COVID-19 patients and retrospective modelling of potential cost savings when administering remdesivir during the pandemic "first wave" in a German tertiary care hospital. Infection. 2022;50(1):191-201.

4. Edoka I, Fraser H, Jamieson L, Meyer-Rath G, Mdewa W. Inpatient Care Costs of COVID-19 in South Africa's Public Healthcare System. International journal of health policy and management.

5. Schwendicke F, Krois J, Gomez J. Impact of SARS-CoV2 (Covid-19) on dental practices: Economic analysis. Journal of dentistry. 2020;99:103387.

6. Lim JT, Dickens BL, Cook AR, Khoo AL, Dan YY, Fisher DA, et al. The costs of an expanded screening criteria for COVID-19: A modelling study. International journal of infectious diseases : IJID : official publication of the International Society for Infectious Diseases. 2020;100:490-6.

7. Hanly P, Ahern M, Sharp L, Ursul D, Loughnane G. The cost of lost productivity due to premature mortality associated with COVID-19: a Pan-European study. European Journal of Health Economics. 2022;23(2):249-59.

8. Chua KP, Conti RM, Becker NV. Trends in and Factors Associated With Out-of-Pocket Spending for COVID-19 Hospitalizations From March 2020 to March 2021. JAMA network open. 2022;5(2):e2148237.

9. Chua KP, Conti RM, Becker NV. Assessment of Out-of-Pocket Spending for COVID-19 Hospitalizations in the US in 2020. JAMA network open. 2021;4(10):e2129894.

10. Viscusi WK. Pricing the global health risks of the COVID-19 pandemic. Journal of Risk and Uncertainty. 2020;61(2):101-28.

11. Garg S, Bebarta KK, Tripathi N, Krishnendhu C. Catastrophic health expenditure due to hospitalisation for COVID-19 treatment in India: findings from a primary survey. BMC research notes. 2022;15(1):86.

12. Faramarzi A, Javan-Noughabi J, Tabatabaee SS, Najafpoor AA, Rezapour A. The lost productivity cost of absenteeism due to COVID-19 in health care workers in Iran: a case study in the hospitals of Mashhad University of Medical Sciences. BMC health services research. 2021;21(1):1169.

13. Viscusi WK. Economic lessons for COVID-19 pandemic policies. Southern Economic Journal. 2021;87(4):1064-89.

14. Chua KP, Conti RM, Becker NV. Out-of-Pocket Spending Within 90 Days of Discharge from COVID-19 Hospitalization. medRxiv : the preprint server for health sciences. 2021.

15. Banke-Thomas A, Makwe CC, Balogun M, Afolabi BB, Alex-Nwangwu TA, Ameh CA. Utilization cost of maternity services for childbirth among pregnant women with coronavirus disease 2019 in Nigeria's epicenter. International journal of gynaecology and obstetrics: the official organ of the International Federation of Gynaecology and Obstetrics. 2021;152(2):242-8.

16. Lopez-Valcarcel BG, Vallejo-Torres L. The costs of COVID-19 and the cost-effectiveness of testing. Applied Economic Analysis. 2021;29(85):77-89.

17. Chua KP, Conti RM, Becker NV. US Insurer Spending on Ivermectin Prescriptions for COVID-19. Jama. 2022;327(6):584-7.

18. Rodriguez-Gonzalez CG, Chamorro-de-Vega E, Valerio M, Amor-Garcia MA, Tejerina F, Sancho-Gonzalez M, et al. COVID-19 in hospitalised patients in Spain: a cohort study in Madrid. International journal of antimicrobial agents. 2021;57(2):106249.

19. Almalki ZS. COVID-19's Economic Impact on the Global Health Systems: Time to Respond to New Realities. Journal of Pharmaceutical Research International. 2021;33(24A):85-91.

20. Nascimento I, Oliveira ALM, Diniz PHC, Leite MF, Oliveira GL. Hospitalization, mortality and public healthcare expenditure in Brazil during the COVID-19 crisis: vulnerabilities in the spotlight. Sao Paulo medical journal = Revista paulista de medicina. 2022;140(2):290-6.

21. Hartman M, Martin AB, Washington B, Catlin A, The National Health Expenditure Accounts T. National Health Care Spending In 2020: Growth Driven By Federal Spending In Response To The COVID-19 Pandemic. Health affairs (Project Hope). 2022;41(1):13-25.

22. Torres-Rueda S, Sweeney S, Bozzani F, Naylor NR, Baker T, Pearson C, et al. Stark choices: exploring health sector costs of policy responses to COVID-19 in low-income and middle-income countries. BMJ global health. 2021;6(12).

23. Fatoye F, Gebrye T, Arije O, Fatoye CT, Onigbinde O, Mbada CE. Economic Impact of COVID-19 lockdown on households. The Pan African medical journal. 2021;40:225.

24. Araja D, Berkis U, Lunga A, Murovska M. Shadow burden of undiagnosed myalgic encephalomyelitis/chronic fatigue syndrome (Me/cfs) on society: Retrospective and prospective—in light of covid-19. Journal of clinical medicine. 2021;10(14).

25. Romero MM, Céspedes AA, Sahuquillo MTT, Zamora EBC, Ballesteros CC, Alfaro VSF, et al. COVID-19 outbreak in long-term care facilities from Spain. Many lessons to learn. PloS one. 2020;15(10 October).

26. Thomas R, Jacob QM, Raj Eliza S, Mini M, Jose J. Financial Burden and Catastrophic Health Expenditure Associated with COVID-19 Hospitalizations in Kerala, South India. ClinicoEconomics and Outcomes Research. 2022:439-46.

27. Chua K-P, Conti RM, Becker NV. Assessment of out-of-pocket spending for COVID-19 hospitalizations in the US in 2020. JAMA network open. 2021;4(10):e2129894-e.

28. Chua K-P, Conti RM, Becker NV. Out-of-pocket spending for health care after COVID-19 hospitalization. The American Journal of Managed Care. 2022;28(8):398-402.
