# Supplementary material for: Costs of inpatient care and out-of-pocket payments for COVID-19 patients: A systematic review
Source: PLoS One. 2023 Sep 20;18(9):e0283651. doi: 10.1371/journal.pone.0283651 (PMC10511135; doi:10.1371/journal.pone.0283651)
Supplement: S6 Table — (DOCX) [file pone.0283651.s007.docx]

**S6 Table. Direct medical costs of inpatients with COVID-19 at Intensive Care Unit (ICU) (Costs were adjusted into Purchasing Power Parity (PPP) 2020)**

| Study ID | Hospitalization day | Treatment | | Diagnostic tests | Hospital bed/day or  Routine Service costs | Others |
| --- | --- | --- | --- | --- | --- | --- |
| Ebrahimipour et al (2022) (1) | 5.7days | Medicine: PPP$ 3114.45  Medical supplies: PPP$ 542.24 | | Radiology: PPP$ 133.75  laboratory tests: PPP$ 207.02 | hoteling: PPP$  3187.66  Visits: PPP$  630.21  Nursing service: PPP$168.86 | Other Costs: PPP$ 143.17 |
| Total Cost: PPP$ 8681.65 | | | | | | |
| ***Popescu et al (2022) (2) | 14.1 days | treatment: PPP$ 3643.09 | | investigation: PPP$ 435.52 |  | administrative: PPP$ 518.5 |
| Total costs: PPP$ 5436.77 | | | | | | |
| Li et al (2020) (3) | *16 days | Drug : PPP$ 14944.1  Therapeutics: PPP$ 9676.11 | | Laboratory: PPP$ 6296.12  Radiology: PPP$ 1358.61 | Bed: PPP$ 828.44 |  |
| Total cost: PPP$ 33137.48 | | | | | | |
| **An et al (2022)(4) | 27 days | Treatment: PPP$ 4003.98  Drug: PPP$ 3252.66  Chinese herbal medicine : PPP$  15.49  Surgery: PPP$ 15.68  Basic medical: PPP$ 0.36  Medical supply: PPP$ 3010.18  Chinese patent medicine: PPP$  554.41 | | Clinical laboratory: PPP$ 5830.77 | Bed: PPP$ 2151.76  Nursing: PPP$  170.74  Consultation: PPP$ 159  Medical examination: PPP$ 929.63 | Other: PPP$ 85.07 |
| Total costs: PPP$ 22118.61 | | | | | | |
| Memirie et al (2022)(5) critical disease | 19.2 days | Drug and supply: PPP$ 1284.14 | | Laboratory & diagnostics: PPP$ 211.22 | Personnel: PPP$ 4445.79 | Food: PPP$ 4941.56  Capital: PPP$ 1040.29  Other: PPP$  48.69 |
| Total costs: PPP$ 11971.98 | | | | | | |
| Oksuz et al (2021)(6) | 14.8 days | Drugs: (PPP$) 1757.21  Medical supplies: (PPP$) 1032.44  Interventions: (PPP$)264.75 | | Laboratory tests: (PPP$) 443.71  Imaging tests:(PPP$) 32.41 | Physician: (PPP$)  16.68  Bed:(PPP$)300.13 | Procedural packages: (PPP$)*****  9870.26 |
| Total costs: (PPP$)13717.61 | | | | | | |
| ****Kotwani et al (2021) (7) | 10.37 days | Medicines: PPP$ 2644.88 | | Incurred towards Diagnosis : PPP$ 158.45 | Hospitalization: PPP$ 6339.7 |  |
| Total cost: PPP$ 9143.2 | | | | | | |
| [Jin](https://www.ncbi.nlm.nih.gov/pubmed/?term=Jin%20H%5BAuthor%5D&cauthor=true&cauthor_uid=33551505) et al (2020) severe (8) | 28 days | $\mathrm{Medicines}^{d}= PPP\$$ 11098.89  Treatment for pre-existing conditions: PPP$ 1334.82  Oxygen therapy: PPP$ $108.91 | | ${Identification and diagnosis}^{a}=$PPP$ 81.32${Identification and diagnosis}^{b}=PPP\$$85.94 | ${Inpatient care}^{c}=PPP\$$4716.51 | Follow-up appointment: PPP$ 7.13 |
| Total Cost: PPP$ 17433.68 | | | | | | |
| [Jin](https://www.ncbi.nlm.nih.gov/pubmed/?term=Jin%20H%5BAuthor%5D&cauthor=true&cauthor_uid=33551505) et al (2020) critical (8) | 42 days | $\mathrm{Medicines}^{d}= PPP\$$19434.93  Treatment for pre-existing conditions: PPP$ 1492.63  Tracheostomy and trachea intubation: PPP$ 85.94  $Use of ventilator \left( including muscle relaxants \right):$  PPP$ 8586.89  Extracorporeal membrane oxygenation: PPP$ 5967.36  Artificial kidney: PPP$ 4125.44  Plasma exchange: PPP$ 1183.51 | | ${Identification and diagnosis}^{a}=PPP\$$81.32  ${Identification and diagnosis}^{b}=PPP\$$85.94 | ${Inpatient care}^{c}=PPP\$$9208.59 | Follow-up appointment: PPP$ 7.15 |
| Total Cost: USD 50223.69 | | | | | | |
| ****Barasa et al (2021) (9) |  | Pharmaceuticals (medicines, etc): PPP$  1316.2  Oxygen therapy PPP$ 286.82  Equipment costs (including ventilator) and monitoring in ICU: PPP$ 235.75 | | COVID-19 test: PPP$ 33.19  Other laboratory tests: PPP$ 399.12  Radiology: PPP$ 54.18 | Staffing: PPP$  6412.73 | Accommodation and overheads: PPP$  878.15  Personal protective equipment: PPP$  62349.11 |
| Total cost: PPP$ 13884.55 | | | | | | |
| [Ghaffari Darab](https://www.ncbi.nlm.nih.gov/pubmed/?term=Ghaffari%20Darab%20M%5BAuthor%5D&cauthor=true&cauthor_uid=33573650) et al (2021) (10) | *7 days | Rehabilitation and Dialysis: PPP$  266.76  Drugs and supplies: PPP $ 3541.98 | | Electrography and Laboratory: PPP $  769.85  Imaging: PPP $ 138.84 | Physician Visit Costs: PPP $ 439.14  Nursing services: PPP PPP $  206.69  Consultant and surgeon: PPP $ 178.61  General and Intensive Care Beds: PPP $  4744.73 | Other services: PPP $  62.4 |
| Total cost: PPP $ 10348.26 | | | | | | |
| Di Fusco et al (2021)(11)  With ICU, but without IMV (N=16,496) | 9.6 days |  | |  |  |  |
| Total costs: PPP $ 25688 | | | | | | |
| Di Fusco et al (2021) (11)  With ICU and IMV( N=21,632) | 18.6days |  | |  |  |  |
| Total costs: PPP $ 78245 | | | | | | |
| ****Thant et al (2021) (12) | 11 days | Medicine: PPP $ 4596.65  Medical Commodities (Masks, Gloves, Gowns, Hand sensitizer, etc.): PPP $ 199.66  Medical Equipment (BP cuff, thermometer, glucometer, pulse oximeter, syringe pump, etc.): PPP $ 68.06  Oxygen therapy: 1810.53  ICU care (Medical Equipment): PPP $ 2727.13 | | Lab investigations per patient (Non-Covid) (CP (auto), urea & electrolytes, Liver/Renal Function tests, etc.): PPP $ 2119.09  COVID-19 Test: PPP $  644.35  Imaging: PPP $95.29 | General HR (Admin staff, general workers, security, etc.): PPP $  313.1 | Direct Contact Health Care Personnel: PPP $  3961.38  PPE: PPP $ 644.35  Non-Medical Equipment per patient (Furniture, computers, generators, etc.): PPP $ 90.75  Center/Hospital Operation per patient (Electricity & Water bill, Maintenance, Meal cost, etc.): PPP $ 1588.18 |
| Total cost: PPP $ 19466.57 | | | | | | |
| [Reddy](https://www.ncbi.nlm.nih.gov/pubmed/?term=Reddy%20KN%5BAuthor%5D&cauthor=true&cauthor_uid=34916743) et al (2021) (13) | 9 days | Surgery Procedure Charges: PPP $ 124.27  Hospital Drugs and Treatment: PPP $ 2486.99  Equipment Charges: PPP $  2113.88 | | Pathology Charges: PPP $  1492.14  Radiological Charges: PPP $  372.94 | Bed Charges: PPP $  2362.55  Nursing Charges: PPP $ 248.67  In-patient Department (IPD) charges: PPP $  3233.08 |  |
| Total cost: PPP $ 12434.86 | | | | | | |
| [Khan](https://www.ncbi.nlm.nih.gov/pubmed/?term=Khan%20AA%5BAuthor%5D&cauthor=true&cauthor_uid=33066327) et al (2020) (14)  Mechanical-Ventilator Use | *7.93 days |  | |  |  |  |
| Total cost: PPP $ 56989.97 | | | | | | |
| [Khan](https://www.ncbi.nlm.nih.gov/pubmed/?term=Khan%20AA%5BAuthor%5D&cauthor=true&cauthor_uid=33066327) et al (2020) (14)  Non Mechanical-Ventilator Use | *7.93 days |  | |  |  |  |
| Total cost: PPP $ 49620.56 | | | | | | |
| Miethke-Morais et al (2021) (15) | 13.93 days | Drugs: PPP $ 1698.59  Supplies: PPP $ 1292.85 | Laboratory Tests: PPP $ 952.45  Radiologic Exams: PPP $ 99.35  Blood components: PPP $546.29 | | Nonmedical staff: PPP $ 20383.18  Medical: PPP $  13007.27  Daily Fixed Costs: PPP $ 2920.64 | PPE: PPP $  848.01  Nutrition: PPP $  114.88 |
| Total cost: PPP $ 41864.14 | | | | | | |
| Gedik (2020) (16) | 14.74 days |  |  | |  |  |
| Total cost: PPP $ 5646.24 | | | | | | |
| Ohsfeldt et al(2021)(17) | 5 days |  |  | |  |  |
| Total costs: PPP $ 13,443 | | | | | | |
| Tsai et al (2021) (18) | 17.1 days |  |  | |  |  |
| Total costs: PPP $ 49441 | | | | | | |
| Schallner et al. (19) | 16 (12–34 [1–85]) days,  median (IQR  [range]) |  |  | |  |  |
| Total cost: PPP $ 100,789 | | | | | | |
| Alvis-Zakzuk et al. (20)  1320.10 | 8.1 (66.7) days | Drugs: PPP $ 978.67  Consults: PPP $ 206.64  Emergencies: PPP $ 117.67  Procedures: PPP $ 63.14 | Diagnostic support: PPP $ 700.28 | | Daily Fixed Costs: PPP $ 4445.63 | 43.05 |
| Total costs: PPP $ 6552.21 | | | | | | |

Data of costs are presented as mean per patient, except for An et al (2022), Kotwani et al (2021), Popescu et al (2022), Ohsfeldt et al (2021), which They presented as median per patient. And in Barasa et al (2020) and [Jin](https://www.ncbi.nlm.nih.gov/pubmed/?term=Jin%20H%5BAuthor%5D&cauthor=true&cauthor_uid=33551505) et al (2021)which They presented as unit cost.

Data of Hospitalization days are presented as mean length of stay or hospitalization days except for Li et al (2020), Reddy et al (2021), Khan et al (2020), Ohsfeldt et al (2021) which They presented as median Hospitalization days.

* Length of Stay Hospitalization for each COVID-19 patient (Ward or ICU)

** Hospitalization days is Average No for Each facility.

***In this paper, due to the small statistical population, the median costs were considered for each component that the sum of the cost component numbers was not equal to the reported cost number.

****In this article, the total cost of components per person was not equal to the total cost per person. Reported number The total cost per person is the number reported by the article.

a Identified from close contacts, b Identified from suspected cases, c Hospital bed days, nursing, blood gas analyses and laboratory tests, d Anti-infective medicines and nutrition support.

*****Including the service package cost included in the reimbursement during the pandemic

ICU: Intensive Care Unit
