# Supplementary material for: Costs of inpatient care and out-of-pocket payments for COVID-19 patients: A systematic review
Source: PLoS One. 2023 Sep 20;18(9):e0283651. doi: 10.1371/journal.pone.0283651 (PMC10511135; doi:10.1371/journal.pone.0283651)
Supplement: S7 Table — (DOCX) [file pone.0283651.s008.docx]

**S7 Table. Direct medical costs of inpatients with COVID-19 at Ward (Costs were adjusted into Purchasing Power Parity (PPP) 2020)**

| Study ID | Hospitalization days | Treatment | | Diagnostic tests | Hospital bed/day or Routine Service costs | Others |
| --- | --- | --- | --- | --- | --- | --- |
| Ebrahimipour et al (2022) (1) | 6.1days | Medicine: PPP$ 588.39  Medical supplies: PPP$ 102.43 | | Radiology: PPP$ 39.11  laboratory tests: PPP$ 129.81 | hoteling: PPP$ 602.2  Visits: PPP$ 119.02  Nursing service: PPP$ 31.88 | Other Costs: PPP$ 27.04 |
| Total cost: PPP$1640.27 | | | | | | |
| Li et al (2020)(3)  Mild | *16 days | Drug: PPP$ 4085.37  Therapeutic measures: PPP$ 2645.07 | | Laboratory: PPP$ 1721.08  Radiology: PPP$ 371.37 | Bed cost: PPP$ 226.46 |  |
| Total Cost: PPP$ 9058.48 | | | | | | |
| Li et al (2020)(3)  Severe | *16 days | Drug: PPP$  9924.43  Therapeutic measures: PPP$ 86025.71 | | Laboratory: PPP$ 4181.02  Radiology: PPP$ 902.22 | Bed: PPP$ 550.13 |  |
| Total Severe Cost: PPP$ 22005.42 | | | | | | |
| ****An et al (2022)  (4) | 18 days | Treatment: PPP$  335.06  Drug: PPP$ 703.72  Chinese herbal medicine: PPP$ 13.46  Surgery: PPP$ 5.19  Basic medical: PPP$  0.28  Medical supply: PPP$  774.09  Chinese patent medicine : PPP$  56.68 | | Clinical laboratory: PPP$ 1004.78 | Bed: PPP$ 389.87  Nursing: PPP$ 123.88  Consultation: PPP$  103.21  Medical examination: PPP$ 398.4 | Other: PPP$ 54.39 |
| Total costs: PPP$ 4208.15 | | | | | | |
| Memirie et al (2022) (5)moderate | 9.2 days | Drug and supply costs: PPP$ 616.42 | | Laboratory & diagnostics: PPP$ 101.35 | Personnel: PPP$  2134.37 | Food: PPP$  2372.37  Capital: PPP$ 499.4  Other: PPP$  23.38 |
| Total costs: PPP$ 5747.64 | | | | | | |
| Memirie et al (2022) (5)severe | 11.3 days | Drug and supply: PPP$  752.28 | | Laboratory & diagnostics: PPP$  123.71 | Personnel: PPP$  2604.71 | Food: PPP$  2895.16  Capital: PPP$  609.49  Other: PPP$  28.49 |
| Total costs: PPP$ 7014.3 | | | | | | |
| Oksuz et al (6)(2021) | 8 days | Drugs: PPP$ 298.32  Medical supplies: PPP$ 18.3 | | Laboratory tests: PPP$ 518.36  Imaging tests: PPP$ 35.27  Interventions: PPP$ 237.77 | Physician: PPP$  9.81  Bed: PPP$ 407.95 | Procedural package ***** PPP$ 2236.39 |
| Total direct costs: PPP$ 3762.31 | | | | | | |
| ****Kotwani et al (2021)(7)  (CCC) | 8.84 days | Medicines patients:  PPP$ 7.88 | | incurred towards Diagnosis patients: PPP$ 145.27 | Hospitalization: PPP$  4226.56 |  |
| Total cost: PPP$ 4379.75 | | | | | | |
| [Jin](https://www.ncbi.nlm.nih.gov/pubmed/?term=Jin%20H%5BAuthor%5D&cauthor=true&cauthor_uid=33551505) et al (2020) (8) | 14 days | $\mathrm{Medicines}^{d}= PPP\$$109.4  Treatment for pre-existing conditions: PPP$ 10.86 | | ${Identification and diagnosis}^{a}=PPP\$$ 81.32  ${Identification and diagnosis}^{b}=PPP\$$87.12 | ${Inpatient care}^{c}=PPP\$$ 1549.11 | Follow-up appointmen: PPP$ 7.13 |
| Total Cost: PPP$ 1845.01 | | | |  |  | |
| [Ghaffari Darab](https://www.ncbi.nlm.nih.gov/pubmed/?term=Ghaffari%20Darab%20M%5BAuthor%5D&cauthor=true&cauthor_uid=33573650) et al (10)(2021) non-sever patient | *7days | Rehabilitation and Dialysis: PPP$ 17.94  Drugs and supplies: PPP$  613.07 | | Electrography and Laboratory: PPP$ 233.99  Imaging: PPP$ 67.85 | Physician Visit Costs: PPP$ 347.87  Nursing services: PPP$  49.91  Consultant and surgeon: PPP$ 46.79  General and Intensive Care Beds: PPP$ 913.38 | Other services: PPP$ 31.19 |
| Total Cost: PPP$ 2323.62 | | | | | | |
| Di Fusco et al (2021)(11)  Without ICU, but with IMV (N=7,751) | 12.1days |  | |  |  |  |
| Total costs: PPP$ 41769 | | | | | | |
| Di Fusco et al (2021) (11)Without ICU or IMV (N=128,063) | 6.1days |  |  | |  |  |
| Total Hospital costs: PPP$ 14325 | | | | | | |
| ****Thant et al (2021)(12) | **11 days | Medicine: PPP$ 4.53  Medical Commodities (Masks, Gloves, Gowns, Hand senstitizer, etc.): PPP$ 199.34  Medical Equipment (BP cuff, thermometer, glucometer, pulse oximeter, syringe pump, etc.): PPP$ 67.96  Oxygen therapy: PPP$ 45.3 | | COVID-19 Test: PPP$ 643.33  Imaging: PPP$ 95.14  Lab investigations (Non-Covid) (CP(auto), urea &electrolytes, Liver/Renal Function tests, etc.): PPP$ 443.99 | General HR (Admin staff, general workers, security, etc.): PPP$ 312.6 | Direct Contact Health Care Personnel per patient: PPP$ 176.69  PPE: PPP$ 643.33  Cost for Non-Medical Equipment per patient (Furniture, computers, generators, etc.): PPP$ 90.61  Center/Hospital Operation (Electricity & Water bill, Maintenance, Meal cost, etc.): PPP$ 1209.64 |
| Total costs: PPP$ 3937.01 | | | | | | |
| [Khan](https://www.ncbi.nlm.nih.gov/pubmed/?term=Khan%20AA%5BAuthor%5D&cauthor=true&cauthor_uid=33066327) et al (2020)(14)  Mechanical-Ventilator Use | *7.93 days |  | |  |  |  |
| Total costs: PPP$ 28814.04 | | | | | | |
| Khan et al (2020)(14)  (Non Mechanical-Ventilator Use) | *7.93 days |  | |  |  |  |
| Total costs: PPP$ 28918.48 | | | | | | |
| [Miethke-Morais](https://www.ncbi.nlm.nih.gov/pubmed/?term=Miethke-Morais%20A%5BAuthor%5D&cauthor=true&cauthor_uid=34454894) et al (2021) (15) | 7.71 | Drugs: PPP$ 364.07  Supplies: PPP$ 320.98 | | Laboratory Tests: PPP$ 241.52  Radiologic Exams: PPP$ 92.84  Blood components: PPP$ 133.46 | Nonmedical staff: PPP$  7636.48  Medical staff: PPP$  3048.33  Daily Fixed Costs: PPP$  849.53 | PPE: PPP$  396.66  Nutrition: PPP$  9.5 |
| Total cost: PPP$ 13093.43 | | | | | | |
| ********Barasa et al (2020) (9) |  | Pharmaceuticals (medicines, etc): PPP$  1010.31  Non- pharmaceuticals (fluids, devices, etc): PPP$ 45.50  Oxygen therapy: PPP$ 245.4 | | COVID-19 test: PPP$ 33.2  Other laboratory tests PPP$ 197.92  Radiology: PPP$ 54.18 | Staffing: PPP$ 362.89 | Accommodation and overheads: PPP$ 417.65  Personal protective equipment: PPP$  810.31 |
| Total cost: PPP$ 2884.15 | | | | | | |
| Gedik (2020) (16) | 8.97days |  | |  |  |  |
| Total cost: PPP$ 1702.66 | | | | | | |
| Tsai et al (2021) (18) | 9.2 days |  | |  |  |  |
| Total cost: PPP$ 21752 | | | | | | |
