# Supplementary material for: Costs of inpatient care and out-of-pocket payments for COVID-19 patients: A systematic review
Source: PLoS One. 2023 Sep 20;18(9):e0283651. doi: 10.1371/journal.pone.0283651 (PMC10511135; doi:10.1371/journal.pone.0283651)
Supplement: S8 Table — (DOCX) [file pone.0283651.s009.docx]

**S8 Table. Combined Direct medical costs of inpatients with COVID-19 at Ward (Costs were adjusted into Purchasing Power Parity (PPP) 2020)**

| Study ID | Hospitalization days | Treatment | Diagnostic | Hospital bed/day or  Routine Service costs | Others |
| --- | --- | --- | --- | --- | --- |
| Yusefi et al (2022)(21) | 6.64 days | Pharmaceutical: PPP $ 4238.57  Rehabilitation: PPP $ 42.5  Material consumable: PPP $ 257.5 | CT scans: PPP $ 84.5  Radiography: PPP $ 41.5  Laboratory: PPP $ 732.76  Radiology: PPP $ 18  Ultrasound: PPP $6.5  Echo: PPP $ 18 | Hoteling (regular bed): PPP$ 1290.77  Hoteling (special bed): PPP $ 1544.02  Visit: PPP $ 742.01  Nursing: PPP $ 143.5  Counseling: PPP $ 104.75 | Other services (transport, Food): PPP $ 109.75 |
| Total cost: PPP $ 9399.4 | | | | | |
| Tabuñar et al (2021) (22)Charity Ward |  | Pharmacy: PPP $ 2128.11  Dialysis: PPP $ 963.42  OR fee: PPP $ 337.62  Cobalt: PPP $ 756.99  Rad Onco: PPP $ 885.17  Ambulance Conduction: PPP $  75.38 | Radiology: PPP $ 180.1  Laboratory: PPP $ 1982.14  ECG: PPP $ 130.42  Ultrasound: PPP $ 10.97  Radio Immunoassay Lab: PPP $  20.85  2D-Echo: PPP $ 81.31  MRI: PPP $ 267.27  MRL: PPP $ 370.84  EMG: PPP $ 159.88  Hearing Screenin: PPP $ 209.94  Central Endoscopy Unit: PPP $  370.2  Cath Lab: PPP $ 383.59  EEG: PPP $ 151.08  Surgical Pathology: PPP $ 9.82  2D-Echo: PPP $ 81.37  Blood Bank: PPP $ 174.87 | Room type: PPP $ 165.81  Doctor's Fee: PPP $ 4454.56  Ventilator Outsource: PPP $  896.14  Central ICU: PPP $ 504.25 | PPE: PPP $ 2052.73 |
| Total cost: PPP $ 17805.57 | | | | | |
| Tabuñar et al (2021) (22)  Pay Ward |  | Pharmacy: PPP $ 2130.54  Oxygen: PPP $ 688.11  Dialysis: PPP $ 1893.56  OR fee: PPP $ 180.61  Cobalt: PPP $ 641.69  Rehab: PPP $ 233.28  Ambulance Conduction: PPP $ 122.49 | Radiology: PPP $ 250.88  Laboratory: PPP $ 2736.89  ECG: PPP $ 52.17  Ultrasound: PPP $ 68.36  Radio Immunoassay Lab: PPP $  45.6  2D-Echo: PPP $ 177.54  Colonoscopy: PPP $ 398.58  Surgical Pathology: PPP $ 33.35  Admission Kit: PPP $ 97.13  Medical Research Lab: PPP $ 340.1  Blood Bank: PPP $ 1036.95 | Room type: PPP $ 1313.73  Doctor's Fee: PPP $ 5031.2  Ventilator Outsource: PPP $ 380.22  Dietary: PPP $ 187.43  Central ICU: PPP $ 435.95 | PPE: PPP $ 1855.55  Reader's fee: PPP $  110.14  Abstract: PPP $4.72 |
| Total cost: PPP $ 20447.44 | | | | | |
| Santos et al (2021) (23) |  |  |  | Hospital services: PPP $  1948.78 | Professional services: PPP $ 351.08 |
| Total cost: PPP $ 2299.89 | | | | | |
| Nakhaei et al (2021) (24) |  | Medicine: PPP$ 373.8 | Diagnostic: PPP$ 277.63 | Visits: PPP$ 287.69  Nursing service: PPP$ 36.93  Hoteling: PPP$ 615.43 | Other: PPP$ 109.24 |
| Total cost: PPP$ 1700.76 | | | | | |
| [Maltezou](https://www.ncbi.nlm.nih.gov/pubmed/?term=Maltezou%20H%5BAuthor%5D&cauthor=true&cauthor_uid=33894306) et al (2021) (25) |  | Hospitalization (Treatment): PPP $ 84.56 | RT-PCR for SARS-CoV-2: PPP $  574.52  Chest radiograph and CT: PPP $  112.21  Biochemistry, complete blood count and urine tests: PPP $  7.12 | Hospitalization: PPP $ 4740.22  ICU stay: PPP $ 1004.99 | Post-discharge: PPP $ 4.43 |
| Total cost: USD 6531.83 | | | | | |
| [Carrera-Hueso](https://www.ncbi.nlm.nih.gov/pubmed/?term=Carrera-Hueso%20FJ%5BAuthor%5D&cauthor=true&cauthor_uid=34734323) et al (2021)(26) | 8 days | Drug: PPP $ 514.78 | Imaging: PPP $ 772.72  Test: PPP $ 1085.93 | Medical visits: PPP $ 36331.76  Nursing hours: PPP $  37320.49  Medical visits, ICU: PPP $  634.69  Nursing hours, ICU: PPP $  1564.72  Hospitalization stays: PPP $  9618.09  ICU stays§§: PPP $  2842.21 |  |
| Total cost: PPP $ 90685.51 | | | | | |
| Hamidi Parsa et al (2021) (27) |  | Medicine and Medical Supplies: PPP $ 8892.77 |  | Personnel costs: PPP $ 43917.62 | Administrative, health and treatment facilities costs: PPP $ 9319.21  Urban amenities costs: PPP $ 842.35  Building depreciation and physical spaces costs: PPP $  3723.29  Other: PPP $  896.46 |
| Total cost: PPP $ 67593.6 | | | | | |
| Haji Aghajani et al (2021) (28) |  | Drug: PPP $ 303.07 | Laboratory Services: PPP $ 470.25  Imaging Services: PPP $ 62.58 | Hoteling and Nursing Services: PPP $ 978.94  Medical services: PPP $ 359.39 |  |
| Total cost: PPP $ 2275.25 | | | | | |
| Haji Aghajani et al (2021)(28) |  | Drug: PPP $ 759.9 | Laboratory Services: PPP $ 639.21  Imaging Services: PPP $ 98.34 | Hoteling and Nursing Services: PPP $ 978.04  Medical services: PPP $ 643.68 |  |
| Total cost: PPP $ 3195.911741 | | | | | |
| Damiri et al( 2021) (29) | *1-10 days | Medicines and Consumable Medical Supplies: PPP $  455.79 | Diagnostic Services: PPP $ 165.88 | Hoteling and Nursing: PPP $  830.98  Visit and Consultation: PPP $ 135.37 | Other services: PPP $ 48.12 |
| Total cost: PPP $ 1636.93 | | | | | |
| Khandehroo et al (2022)-Iran | 5 days (Median) | Drugs and supplies: PPP $ 1096.51  Operation Room &  Anesthesia: PPP $ 29.63  Visits & Consultations: PPP $ 355.63 | Laboratory and Diagnostic  Test: PPP $ 296.35  Imaging Services: PPP $  88.91 | Hoteling & Nursing  Services: PPP $ 918.7005 | Other services: PPP $ 177.813 |
| Total cost: PPP $ 2963.55 | | | | | |
| Forrest et al (2021)-USA | 4.8 days |  |  |  |  |
| Total cost: PPP $ 17103 | | | | | |

Data of costs are presented as mean per patient, except for Haji Aghajani et al (2021) presented as median cost per patient.

Data of Hospitalization days are presented as mean length of stay or hospitalization days in Yusefi et al (2022) but in Carrera-Hueso et al (2021) presented as median Length of Stay Hospitalization for each COVID-19 patient (Ward or ICU) and in Damiri et al (2021) the majority of patients (87.3%) had a stay length of less than 10 days,

* Length of stay of 87.3% patients.

§§ In the ICU, daily blood test and blood gas analysis

References:

1. Ebrahimipour H, Haghparast-Bidgoli H, Aval SB, Hoseini SJ, Jamili S, Ebnehoseini Z, et al. Diagnostic and Therapeutic Costs of Patients With a Diagnosis of or Suspected Coronavirus Disease in Iran. Value in health regional issues. 2022;27:21-4.

2. Popescu M, Ştefan OM, Ştefan M, Văleanu L, Tomescu D. ICU-Associated Costs During The Fourth Wave Of The Covid-19 Pandemic In A Tertiary Hospital In A Low-Vaccinated Eastern European Country. International journal of environmental research and public health. 2022;19(3).

3. Li XZ, Jin F, Zhang JG, Deng YF, Shu W, Qin JM, et al. Treatment of coronavirus disease 2019 in Shandong, China: a cost and affordability analysis. Infectious diseases of poverty. 2020;9(1):78.

4. An X, Xiao L, Yang X, Tang X, Lai F, Liang XH. Economic Burden Of Public Health Care And Hospitalisation Associated With Covid-19 In China. Public Health. 2022;203:65-74.

5. Memirie ST, Yigezu A, Zewdie SA, Mirkuzie AH, Bolongaita S, Verguet S. Hospitalization Costs For Covid-19 In Ethiopia: Empirical Data And Analysis From Addis Ababa's Largest Dedicated Treatment Center. PLoS One. 2022;17(1):e0260930.

6. Oksuz E, Malhan S, Gonen MS, Kutlubay Z, Keskindemirci Y, Tabak F. Covid-19 Healthcare Cost And Length Of Hospital Stay In Turkey: Retrospective Analysis From The First Peak Of The Pandemic. Health economics review. 2021;11(1):39.

7. Kotwani P, Patwardhan V, Pandya A, Saha S, Patel GM, Jaiswal S, et al. Valuing Out-Of-Pocket Expenditure And Health Related Quality Of Life Of Covid-19 Patients From Gujarat, India. Journal of Communicable Diseases. 2021;53(1):104-9.

8. Jin H, Wang H, Li X, Zheng W, Ye S, Zhang S, et al. Economic Burden Of Covid-19, China, January–March, 2020: A Cost-Of-Illness Study. Bulletin of the World Health Organization. 2021;99(2):112-24.

9. Barasa E, Kairu A, Maritim M, Were V, Akech S, Mwangangi M. Examining unit costs for COVID-19 case management in Kenya. BMJ global health. 2021;6(4):e004159.

10. Ghaffari Darab M, Keshavarz K, Sadeghi E, Shahmohamadi J, Kavosi Z. The Economic Burden Of Coronavirus Disease 2019 (Covid-19): Evidence From Iran. BMC health services research. 2021;21(1):132.

11. Di Fusco M, Shea KM, Lin J, Nguyen JL, Angulo FJ, Benigno M, et al. Health outcomes and economic burden of hospitalized COVID-19 patients in the United States. Journal of Medical Economics. 2021;24(1):308-17.

12. Thant PW, Htet KT, Win WY, Htwe YM, Htoo TS. Cost Estimates Of Covid-19 Clinical Management In Myanmar. BMC health services research. 2021;21(1):1365.

13. Reddy KN, Shah J, Iyer S, Chowdhury M, Yerrapalem N, Pasalkar N, et al. Direct Medical Cost Analysis of Indian COVID-19 Patients Requiring Critical Care Admission. Indian Journal of Critical Care Medicine: Peer-reviewed, Official Publication of Indian Society of Critical Care Medicine. 2021;25(10):1120.

14. Khan AA, AlRuthia Y, Balkhi B, Alghadeer SM, Temsah M-H, Althunayyan SM, et al. Survival And Estimation Of Direct Medical Costs Of Hospitalized Covid-19 Patients In The Kingdom Of Saudi Arabia. International journal of environmental research and public health. 2020;17(20):7458.

15. Miethke-Morais A, Cassenote A, Piva H, Tokunaga E, Cobello V, Gonçalves FAR, et al. Covid-19-Related Hospital Cost-Outcome Analysis: The Impact Of Clinical And Demographic Factors. Brazilian Journal of Infectious Diseases. 2021;25.

16. Gedik H. The Cost Analysis Of Inpatients With Covid-19. Acta Medica Mediterr. 2020;36(1):3289-92.

17. Ohsfeldt RL, Choong CK-C, Mc Collam PL, Abedtash H, Kelton KA, Burge R. Inpatient Hospital Costs For Covid-19 Patients In The United States. Advances in therapy. 2021;38(11):5557-95.

18. Tsai Y, Vogt TM, Zhou F. Patient Characteristics And Costs Associated With Covid-19–Related Medical Care Among Medicare Fee-For-Service Beneficiaries. Annals of internal medicine. 2021;174(8):1101-9.

19. Schallner N, Lieberum J, Kalbhenn J, Bürkle H, Daumann F. Intensive care unit resources and patient‐centred outcomes in severe COVID‐19: a prospective single‐centre economic evaluation. Anaesthesia. 2022;77(12):1336-45.

20. Alvis-Zakzuk NJ, Flórez-Tanus Á, Díaz-Jiménez D, Chaparro-Narváez P, Castañeda-Orjuela C, De La Hoz-Restrepo F, et al. How Expensive Are Hospitalizations by COVID-19? Evidence From Colombia. Value in health regional issues. 2022;31:127-33.

21. Yusefi AR, Mehralian G, Khodamoradi A, Abbasi R, Vatankhah F, Heaidari F, et al. Out-Of-Pocket Payments For Treatment Of Covid-19 In Iran. Cost effectiveness and resource allocation : C/E. 2022;20(1):1-10.

22. Tabuñar SMS, Dominado TMP. Hospitalization Expenditure Of Covid-19 Patients At The University Of The Philippines-Philippine General Hospital (Up-Pgh) With Philhealth Coverage. Acta Medica Philippina. 2021;55(2):216-23.

23. Santos H, Maciel FBM, Santos Junior GM, Martins PC, Prado N. Public Expenditure On Hospitalizations For Covid-19 Treatment In 2020, In Brazil. Revista de saude publica. 2021;55:1-11.

24. Nakhaei K, Jalilian H, Arab-Zozani M, Heydari S, Torkzadeh L, Taji M. Direct and indirect cost of COVID-19 patients in Iran. Health policy and technology. 2021;10(4):100572.

25. Maltezou HC, Giannouchos TV, Pavli A, Tsonou P, Dedoukou X, Tseroni M, et al. Costs Associated With Covid-19 In Healthcare Personnel In Greece: A Cost-Of-Illness Analysis. The Journal of hospital infection. 2021;114:126-33.

26. Carrera-Hueso FJ, Álvarez-Arroyo L, Poquet-Jornet JE, Vázquez-Ferreiro P, Martínez-Gonzalbez R, El-Qutob D, et al. Hospitalization Budget Impact During The Covid-19 Pandemic In Spain. Health economics review. 2021;11(1):43.

27. Parsa HH, Saghafipour A, Koohpaei A, Farzinnia B, Barouni M. In-Hospital Economic Burden for COVID-19 Infection Using Step-Down Cost Accounting; a Case from Central Iran. Shiraz E Medical Journal. 2022;23(2).

28. Aghajani MH, Sistanizad M, Toloui A, Neishaboori AM, Pourhoseingholi A, Maher A, et al. Covid-19 Related Hospitalization Costs; Assessment Of Influencing Factors. Frontiers in Emergency Medicine. 2022;6(1):e3-e.

29. Damiri S, Nahvijou A, Sargazi N, Fazaeli AA, Akbari Sari A, Daroudi R. Hospitalization costs of patients with Covid-19: A study in Tehran University of Medical Sciences. Health Management & Information Science. 2021;8(3):168-76.
