# Supplementary material for: Costs of inpatient care and out-of-pocket payments for COVID-19 patients: A systematic review
Source: PLoS One. 2023 Sep 20;18(9):e0283651. doi: 10.1371/journal.pone.0283651 (PMC10511135; doi:10.1371/journal.pone.0283651)
Supplement: S9 Table — (DOCX) [file pone.0283651.s010.docx]

**S9 Table. COVID-19 hospitalization Cost diver in the included study**

| **Study** | **Variables** |
| --- | --- |
| Nakhaei et al (2021)(1) | Type of Insurance |
| Nakhaei et al (2021)(1), Li et al (2020)(2), Liang et al (2022)(3), Oksuz et al (2021)(4), Miethke-Morais et al (2021)(5), Tsai et al (2021)(6) | Age group |
| Liang et al (2022)(3), Miethke-Morais et al (2021)(5), Damiri et al( 2021)(7) | Length of stay |
| Liang et al (2022)(3) | Hospitalization in the Negative-pressure isolation ward (NPIW ) |
| Liang et al (2022)(3), Reddy et al (2021)(8), Oksuz et al (2021)(4) | use of non-invasive ventilation |
| Liang et al (2022)(3), Ohsfeldt et al (2021)(9), Damiri et al( 2021)(7) | admission to the ICU |
| Ebrahimipour et al (2022), Popescu et al (2022), Li et al (2020)(2), Liang et al (2022)(3), Oksuz et al (2021)(4), Reddy et al (2021)(8), | disease severity |
| Liang et al (2022)(3) | Number of hospitalizations |
| Popescu et al (2022)(10) | Treatment with antiviral immunomodulatory drugs |
| Nakhaei et al (2021)(1), Oksuz et al (2021)(4) | Die |
| Li et al (2020)(2), Oksuz et al (2021)(4), Reddy et al (2021)(8), Miethke-Morais et al (2021)(5), Ohsfeldt et al (2021)(9), Haji Aghajani et al (2021)(11), Damiri et al(2021)(7) | Pre-existing diseases |
| Reddy et al (2021)(8) | Median delay of ≥6 days in ICU admission from symptom onset |
| Reddy et al (2021)(8) | Length of stay in ICU |
| Miethke-Morais et al (2021)(5), Tsai et al (2021)(6), Ohsfeldt et al (2021)(9) | Sex |
| Miethke-Morais et al (2021)(5), Reddy et al (2021) | Mechanical ventilation |
| Miethke-Morais et al (2021)(5), Popescu et al (2022)(10), Haji Aghajani et al (2021)(11) | Dialysis |
| Miethke-Morais et al (2021)(5) | Surgery |
| Miethke-Morais et al (2021)(5) | Transfer |
| Tsai et al (2021)(6) | Patients in urban counties |
| Ohsfeldt et al (2021)(9) | Ethnicity |
| Haji Aghajani et al (2021)(11) | New cardiovascular complications |
| Haji Aghajani et al (2021)(11) | History of drug abuse |
| Haji Aghajani et al (2021)(11) | Pregnancy |

**References:**

1. Nakhaei K, Jalilian H, Arab-Zozani M, Heydari S, Torkzadeh L, Taji M. Direct and indirect cost of COVID-19 patients in Iran. Health policy and technology. 2021;10(4):100572.

2. Li XZ, Jin F, Zhang JG, Deng YF, Shu W, Qin JM, et al. Treatment of coronavirus disease 2019 in Shandong, China: a cost and affordability analysis. Infectious diseases of poverty. 2020;9(1):78.

3. An X, Xiao L, Yang X, Tang X, Lai F, Liang XH. Economic Burden Of Public Health Care And Hospitalisation Associated With Covid-19 In China. Public Health. 2022;203:65-74.

4. Oksuz E, Malhan S, Gonen MS, Kutlubay Z, Keskindemirci Y, Tabak F. Covid-19 Healthcare Cost And Length Of Hospital Stay In Turkey: Retrospective Analysis From The First Peak Of The Pandemic. Health economics review. 2021;11(1):39.

5. Miethke-Morais A, Cassenote A, Piva H, Tokunaga E, Cobello V, Gonçalves FAR, et al. Covid-19-Related Hospital Cost-Outcome Analysis: The Impact Of Clinical And Demographic Factors. Brazilian Journal of Infectious Diseases. 2021;25.

6. Tsai Y, Vogt TM, Zhou F. Patient Characteristics And Costs Associated With Covid-19–Related Medical Care Among Medicare Fee-For-Service Beneficiaries. Annals of internal medicine. 2021;174(8):1101-9.

7. Damiri S, Nahvijou A, Sargazi N, Fazaeli AA, Akbari Sari A, Daroudi R. Hospitalization costs of patients with Covid-19: A study in Tehran University of Medical Sciences. Health Management & Information Science. 2021;8(3):168-76.

8. Reddy KN, Shah J, Iyer S, Chowdhury M, Yerrapalem N, Pasalkar N, et al. Direct Medical Cost Analysis of Indian COVID-19 Patients Requiring Critical Care Admission. Indian Journal of Critical Care Medicine: Peer-reviewed, Official Publication of Indian Society of Critical Care Medicine. 2021;25(10):1120.

9. Ohsfeldt RL, Choong CK-C, Mc Collam PL, Abedtash H, Kelton KA, Burge R. Inpatient Hospital Costs For Covid-19 Patients In The United States. Advances in therapy. 2021;38(11):5557-95.

10. Popescu M, Ştefan OM, Ştefan M, Văleanu L, Tomescu D. ICU-Associated Costs During The Fourth Wave Of The Covid-19 Pandemic In A Tertiary Hospital In A Low-Vaccinated Eastern European Country. International journal of environmental research and public health. 2022;19(3).

11. Aghajani MH, Sistanizad M, Toloui A, Neishaboori AM, Pourhoseingholi A, Maher A, et al. Covid-19 Related Hospitalization Costs; Assessment Of Influencing Factors. Frontiers in Emergency Medicine. 2022;6(1):e3-e.
